# Supplementary material for: Integrative inference of subclonal tumour evolution from single-cell and bulk sequencing data
Source: Nat Commun. 2019 Jun 21;10:2750. doi: 10.1038/s41467-019-10737-5 (PMC6588593; doi:10.1038/s41467-019-10737-5)
Supplement: Supplementary file 1 — Supplementary Information [file 41467_2019_10737_MOESM1_ESM.pdf]

Supplementary Information

Integrative inference of subclonal tumour evolution from single-cell and bulk sequencing data

Malikic et al.

A Supplementary Methods

A.1 Generation of simulated datasets

Simulations with 6 and 10 cell populations

Using the notation from Methods, we randomly generated 100 clonal trees of tumour evolution for each $s \in \{5, 9\}$ (i.e. simulated trees are of size 6 or 10 when root node representing population of healthy cells is included). For each tree, we randomly spread 50 mutations to its nodes. In order to account for the population of healthy cells, we did not assign any mutation to the root node. Furthermore, to ensure that each node corresponds to a different subclonal population, unique with respect to the set of mutations it harbours, we assigned at least one mutation to each of the s non-root nodes. While simulating frequencies ϕ_{ij} of cellular populations we set the lower bound for ϕ_{ij} to $\varphi = 0.02$. To simulate values of ϕ_{ij} satisfying this constraint, for a given sample $\mathcal{B}_j$ we first simulated $s + 1$ random numbers $w_{1j}, w_{2j}, \ldots, w_{(s+1)j}$ from the interval $(0, 1)$ and for each $i = 1, 2, \ldots, s + 1$ assigned value ϕ_{ij} as

$$\phi_{ij} = \varphi + [1 - \varphi \cdot (s + 1)] \cdot \frac{w_{ij}}{\sum_{l=1}^{s+1} w_{lj}}.$$

For mutation M with cellular prevalence y in a given bulk sample (that is calculated as the sum of frequencies of cellular populations harbouring M in that sample) bulk sequencing read counts were drawn from binomial distribution with parameters t (number of trials) that accounts for the sequencing coverage and $\frac{y}{2}$ (success probability).

Before sampling single-cell data from the subclone genotypes, we first added sampling distortion to the subclonal frequencies. We assume that the true subclonal frequencies in physical sample used for SCS data sampling are given by the vector $\Psi = [\psi_1, \ldots, \psi_{s+1}]$, where ψ_i equals the average cellular prevalence of subclone v_i among all bulk samples. Sampling distortion is added in order to account for the fact that in practice single cells may not be sampled uniformly from the entire tumour cell population. Due to such sampling biases, it is unlikely that the sampled single-cell genotypes perfectly represent the subclone frequencies in the tumour. Therefore we obtain new (observed) genotype prevalences by sampling from a Dirichlet distribution $\Psi_{observed} \sim Dir(\lambda\Psi)$ following the approach in [1]. The larger the value of λ the smaller is the difference between the observed frequencies and the bulk subclone frequencies.

To obtain the genotypes of the m single-cell samples, we then sampled m times (with replacement) from the s tumour subclone genotypes using the observed genotype prevalences as sampling weights. We then converted each sample with probability δ into a doublet by sampling a second genotype and replacing the sample's original genotype with the union of the two genotypes. The obtained genotypes form a $n \times m$ binary matrix. We then added false positives and false negatives by flipping each 0 in the mutation matrix to a 1 with probability α and each 1 to a 0 with probability $\beta^* = e^z \cdot \beta$, where z is sampled individually for each tree from a normal distribution with standard deviation 0.1 to obtain around 10% misspecification for the false negative rate. Finally, we added missing data points (NA) by converting each matrix entry to NA with probability μ. In all of our simulations we use $\mu = 0.05$.

Only mutations present in at least one of the single cells were retained for analysis, unless stated otherwise. For high distortion rates (low λ) of the single-cell sampling, some mutations may not be observed in any of the sampled single cells. If not retained for the analysis, these mutations are correspondingly removed from the ground truth tree when comparing the accuracy.

Simulations with 20 and 40 cell populations

In order to evaluate the performance of B-SCITE in the presence of a higher degree of intra-tumour heterogeneity we randomly generated 100 trees of tumour evolution for each $s \in \{19, 39\}$. All details of these simulations are same as in Supplementary Section A.1 except that the number of simulated mutations was 100 (this value was motivated by the expectation that an arbitrary subclonal population v_j is likely characterized by multiple mutations occurring for the first time at v_j) and the minimum cell population frequency, denoted above as φ, was set to 0.01 (which allows higher variability in subclonal frequencies).

Simulation of CNA events

In this section we provide details of simulating CNA events and modifications required in simulating bulk and single-cell data in order to account for the presence of CNAs.

For an arbitrary mutation $M \in \mathbf{M}$, occurring at genomic position $\mathrm{pos}(M)$, we randomly choose, with a probability of success equal to a given constant p, whether $\mathrm{pos}(M)$ belongs to a region of genome affected by some CNA event. In case of success, we randomly choose a node $v \neq N_T(M)$ representing the first occurrence of the simulated CNA event and assume that it affects region $\mathcal{R}$ which spans $\mathrm{pos}(M)$. For the sake of simplicity, we assume that it does not span genomic position of any mutation from the set $\mathbf{M} \setminus \{M\}$.

When simulating CNA event we have to distinguish between physical copy of region $\mathcal{R}$ containing reference nucleotide at $\mathrm{pos}(M)$ (i.e. the same nucleotide as contained in the healthy cells) and physical copy containing variant nucleotide (i.e. nucleotide supporting M). We refer to the two as reference and variant copy of region $\mathcal{R}$, respectively. Distinguishing between reference and variant copies is important in simulating bulk and single-cell data as losses/gains of the two are not equivalent. Thus we represent simulated CNA event as a pair (e, f), where e and f denote the total change (due to this event) in the number of variant and reference copies of $\mathcal{R}$. Note that negative values of e, f represent deletions, whereas positive values represent gains. Due to the above assumptions, we can also conclude that, prior to CNA event affecting region $\mathcal{R}$, each cell has either two reference or one reference and one variant copy of this region.

Since our goal is simulating CNA events undetected during the tumour copy-number profile analysis (which is performed as part of the input data pre-processing) we expect that the events with gains or losses of multiple copies of the same genomic region are successfully detected during this step and therefore restrict ourselves to CNA events such that $|e| + |f| = 1$. In other words, the set of possible CNA events consists of: $(0, -1)$ (loss of reference copy of $\mathcal{R}$), $(0, 1)$ (gain of reference copy of $\mathcal{R}$), $(-1, 0)$ (loss of variant copy of $\mathcal{R}$) and $(1, 0)$ (gain of variant copy of $\mathcal{R}$). If $A_T[N_T(M), v] = 0$ then the simulated CNA event occurs at the subclone which does not harbour M hence in this case the gain or loss of variant copy of $\mathcal{R}$ is not possible and we randomly choose (e, f) from the set $\{(0, 1), (0, -1)\}$. Otherwise, we choose (e, f) randomly from the set $\{(0, -1), (0, 1), (-1, 0), (1, 0)\}$. In each case, each of the candidate events has the same probability to be chosen (i.e. probability is 0.50 for each of the two candidate events in the first and 0.25 for each of the four candidate events in the second case).

Note that the the true variant allele frequency of mutation M_i in bulk sample $\mathcal{B}_j$ can now be calculated by using the following formula:

$$\mathrm{VAF}(M_i)_j = \frac{\sum_{k=1}^{s} \Big(A_T[N_T(M_i), v_k] + e_i A_T[v_j, v_k]\Big)\phi_{kj}}{2\phi_{s+1} + \sum_{k=1}^{s} \Big(2 + (e_i + f_i) A_T[v_j, v_k]\Big)\phi_{kj}}.$$

For simulating bulk data, number of variant reads r_{ij} is drawn from the binomial distribution with parameters t (number of trials) and $\mathrm{VAF}(M_i)_j$ (success probability), where t denotes the coverage of bulk data.

In order to properly simulate SCS data in the cases where CNA event affecting genomic position of mutation M_i represents loss of variant allele, prior to sampling single cells we first update genotypes of subclones corresponding to the node of occurrence of the loss and all of its descendants by assuming that M_i is absent from all of these subclones in the ground truth. SCS data is then simulated analogously as described above for the cases where no CNA events are present.

We generated simulations with $p \in \{0.10, 0.30, 0.50\}$ and varying depth (10,000 and 1,000,000) of bulk data coverage. Results and details of SCS data parameters used in these simulations are presented in Supplementary Figure 18 and Supplementary Figure 19. In each case 100 simulations were generated.

Simulation of mutations violating ISA

In generating simulations containing mutations violating ISA we consider three types of ISA violations. These include: (i) ISA violations due to deletion affecting genomic region harbouring mutation (ii) ISA violations due to loss of heterozygosity (LOH) and (iii) parallel mutations (mutations affecting the same

genomic position, but occurring at subclones which belong to different branches/lineages of tree of tumour evolution). Currently, there is no gold standard for simulating mutations violating ISA and below we provide description of the approach used in this work. Since frequencies of the three types of ISA violations mentioned above are not expected to be equal (e.g. ISA violations due to deletions are expected to be more frequent than parallel mutations), we assign them different weights (see below). In our simulations we also take into account topological differences among trees (e.g. trees having a lot of branching are expected to have a higher number of simulated parallel mutations).

We start by simulating tree without ISA violations using the approach described in Supplementary Section A.1. ISA violations are then added incrementally. Assuming that we have a tree T in which ISA is violated for $b \geq 0$ mutations, we modify this tree in order to obtain tree T', such that the only difference between the two trees is the presence of one additional mutation violating ISA in T' in comparison to T. In order to choose an additional ISA-violating mutation, we first consider the set of all mutations in T for which ISA is not violated. For an arbitrary mutation M from this set, we compute its *ISA-violating-weight* as follows: we iterate over all nodes v of T, excluding the root node and $N_T(M)$ (i.e. over all nodes at which it is possible to introduce mutation that causes violation of ISA for M). If v is on different lineage (branch) in T in comparison to the $N_T(M)$, then the only way to introduce mutation at v that will cause ISA violation of M is via parallel mutation and therefore in this case we increase *ISA-violating-weight* of M by the weight of parallel mutation (here set to 0.1). Otherwise, *ISA-violating-weight* of M is increased by the sum of weights of ISA violations due to deletion and LOH, here set to 0.7 and 0.2, respectively. After computing *ISA-violating-weight* for each mutation for which ISA is not violated in T, we choose one of these mutations proportionally to their *ISA-violating-weight*s and add it to the set of ISA-violating mutations of T. In the rest of this section, we denote the selected mutation by M.

Next, we choose the type of ISA violation for M. First, we decide whether it is parallel mutation or not. To do this, similarly as above, we assign weight to each of the two possibilities. For parallel mutation, we assign weight equal to the product of: (i) 0.1 and (ii) the number of nodes which are on different branches in T in comparison to $N_T(M)$. Weight of the non-parallel ISA violating mutation is set to the product of (i) 0.9(=0.7+0.2) and (ii) the number of non-root nodes which are either ancestors or descendants of $N_T(M)$. Again, proportionally to weights, we choose whether ISA violation is due to parallel mutation or not.

In the case that parallel mutation was chosen above, we choose uniformly at random one of the candidate nodes where parallel mutation might occur and introduce mutation parallel to M at the selected node. Otherwise, we first decide whether ISA violation is due to deletion or LOH by selecting one of the two proportionally to their weights, 0.7 and 0.2, respectively. In either case, we select one of the non-root ancestors or descendants of $N_T(M)$. Here, we have to pay attention to the case where the selected node is ancestor of $N_T(M)$. In such case we swap the roles of the two nodes so that the selected node is the node of the first occurrence of M and then simulate deletion or LOH event at the other node.

After the above steps, we obtain desired tree T' with $b + 1$ ISA-violating mutations. Bulk file and single-cell files are then obtained by updating the corresponding files for tree T. The only updates made are for the read count data in bulk file and in the row of single-cell data matrix which corresponds to M.

In our simulations, the above steps are repeated until the number of ISA-violating mutations reaches 5.

A.2 Phylogenetic accuracy measures

Assuming that T and I represent simulated ground truth and inferred trees, respectively, the three measures used for tree comparison are defined as follows

1. ***Ancestor-descendant accuracy:*** For this accuracy measurement, we consider all pairs of mutations (a, b) that are in ancestor-descendant relation in T. In other words, $A_T[N_T(a), N_T(b)] = 1$ and $N_T(a) \neq N_T(b)$. For each pair, we check whether this dependency between a and b is preserved in I. The total score is defined as number of preserved relations in I among such mutation pairs divided by their total number in T.

2. ***Different-lineage (siblings) accuracy:*** Here we consider only pairs of mutations (a, b) that belong to different lineages in T. In other words $A_T[N_T(a), N_T(b)] = A_T[N_T(b), N_T(a)] = 0$. Analogously to the previous measure, we count the number of times this relation is preserved in I and divide by the total number of pairs of mutations satisfying this relation in T.

3. ***Co-clustering accuracy:*** It is expected that mutations originating from the same subclone in T lie on the same lineage and in close proximity in I. In this measure, for each pair of mutations (a, b) such that $N_T(a) = N_T(b)$ we define the co-clustering score of pair (a, b). If $N_I(a)$ and $N_I(b)$ are not on the same lineage (i.e. $A_I[N_I(a), N_I(b)] = A_I[N_I(b), N_I(a)] = 0$) this score is defined to be equal to 0, otherwise we define it as $\frac{q}{p}$, where p represents the total number of mutations on the path from $N_I(a)$ to $N_I(b)$, including the end-nodes, and q represents number of mutations c on this path such that $N_T(c) = N_T(a)$. The total score of I is defined as the average of the scores over all mutation pairs belonging to the same subclone in T.

Note that in the computation of the above measures for the cases where ground truth tree contains some mutations violating ISA, such mutations are discarded from the measure computation as their phylogenetic (ancestor-descendant vs. different-lineage) relation with the other mutations is not always uniquely identifiable.

A.3 Derivation of the Binomial distribution approximation formula

In this section we provide more detailed derivation of the approximation formula shown in (5).

Assume that we are given a binomial distribution with parameters t (number of trials) and $\frac{y}{2}$ (success probability). Recall that in our case, number of trials represents number of reads, whereas success probability equals to the probability of drawing variant read. In our notation, number of variant reads (i.e. successes) is denoted as r.

It is well known that for sufficiently large value of t, this binomial distribution can be approximated by the Gaussian distribution with mean $\mu = t \cdot \frac{y}{2}$ and standard deviation $\sigma = \sqrt{t \cdot \frac{y}{2} \cdot \left(1 - \frac{y}{2}\right)}$. As the probability density function f of the Gaussian distribution with mean value μ and standard deviation σ is given by

$$f(x \mid \mu, \sigma^2) = \frac{1}{\sqrt{2\pi\sigma^2}} e^{-\frac{(x-\mu)^2}{2\sigma^2}}$$

we have that in our case the probability of observing r variant reads is

$$\mathrm{Binom}\left(r, \frac{y}{2}\right) \approx f(r \mid \mu, \sigma^2) = \frac{1}{\sqrt{2\pi t \cdot \frac{y}{2} \cdot \left(1 - \frac{y}{2}\right)}} e^{-\frac{(r - t \cdot \frac{y}{2})^2}{2t \cdot \frac{y}{2} \cdot \left(1 - \frac{y}{2}\right)}}.$$

The log of the expression in the right-hand side of the above equation equals to

$$\begin{aligned}\log \frac{1}{\sqrt{2\pi t \cdot \frac{y}{2} \cdot \left(1 - \frac{y}{2}\right)}} - \frac{(r - t \cdot \frac{y}{2})^2}{2t \cdot \frac{y}{2} \cdot \left(1 - \frac{y}{2}\right)} &= \log\left(\frac{1}{\sqrt{2\pi}} \cdot \frac{1}{\sqrt{t \cdot \frac{y}{2} \cdot \left(1 - \frac{y}{2}\right)}}\right) - \frac{\left[\frac{t}{2}\left(\frac{2r}{t} - y\right)\right]^2}{2t \cdot \frac{y}{2} \cdot \left(1 - \frac{y}{2}\right)} \\ &= \log \frac{1}{\sqrt{2\pi}} + \log \frac{1}{\sqrt{t \cdot \frac{y}{2} \cdot \left(1 - \frac{y}{2}\right)}} - \frac{t}{8 \cdot \frac{y}{2} \cdot \left(1 - \frac{y}{2}\right)} \cdot (z - y)^2\end{aligned}$$

where in the last step we used $z = \frac{2r}{t}$. This completes the derivation of the expression shown in Equation (5) in the main manuscript.

A.4 Details of running ddClone, OncoNEM, SCITE, PhyloWGS and B-SCITE

In this section we provide a brief description of the input data and the parameter settings used for running B-SCITE and four methods that B-SCITE was compared against. In order to allow fairer comparison with single-cell data based methods OncoNEM and SCITE, we filtered all mutations which are not detected in any single-cell (i.e. their corresponding row in the simulated matrix D consists only of zeroes or missing entries) as these methods have no signal for the placement of such mutations in the tree of tumour evolution. This filtering is applied to both bulk and SCS data. Whenever matrix D is mentioned below, we refer to the matrix obtained after this filtering step.

Details of running ddClone

Since it was unclear to us how missing entries in single-cell data are treated in ddClone, as single-cell data part of the input to this tool we provide matrix D' obtained from D by replacing each of the missing entries with the corresponding true value. ddClone also requires purity value as part of the input. In each case, the true simulated value of purity was provided. Bulk data read counts were also provided as required part of the input. In each case, we run ddClone for 300 iterations. The choice of this value was primarily motivated by the default value set to 100 in the examples published with this tool that are of comparable size to our simulated datasets. In addition, running time of ddClone increases significantly as the number of iterations is further increased.

Details of running OncoNEM

The input to OncoNEM was directly obtained from matrix D after removing cells (i.e. columns) having none of the entries equal to 1, as such cells are assumed to be filtered prior to running this tool. For each run, values of false positive and false negative error rates were given as values of α and β used while simulating matrix D (see A.1). Due to runtime errors, this tool could not terminate properly on several instances with 100 simulated cells and none of the results are shown for these samples in the corresponding accuracy plots.

Details of running SCITE

Matrix D was used as SCS data input to SCITE. Values of false positive and false negative error rates were provided analogously as for OncoNEM. We ran this tool for 3 repeats with 500,000 iterations for each repeat.

Details of running PhyloWGS

Bulk file, in the format specified in the PhyloWGS input data description, was provided as the input to this tool. Number of burn-in and true MCMC samples were respectively set to 2000 and 5000, the values that are 2 times larger than the default ones. In summary, assuming that bulkFile.txt denotes path to the bulk file, we used the following command to run PhyloWGS:

```
python2 multiEvolve.py --num-chains 4 --ssms bulkFile.txt --burnin-samples 2000 --mcmc-samples 5000
```

As PhyloWGS samples large number of trees and reports them in the output, as per recommendations provided at the github repository, we selected a tree with the lowest nlgLH (normalized log likelihood) as the single best tree and used it in the computation of the phylogenetic accuracy measures.

Details of running B-SCITE

Input to B-SCITE consists of bulk data read counts, matrix D and priors for false positive and false negative error rates of SCS data, which were provided analogously as for OncoNEM. We ran B-SCITE for 3 repeats with 500,000 iterations for each repeat.

Here, it is worth mentioning that in the implementation of the accompanying software, we allow the option of setting parameter w which controls weights of bulk and SCS data likelihoods in the joint likelihood calculation. More precisely, we consider the joint likelihood $2\,[wS_{\text{sc}}(T, \boldsymbol{\theta}) + (1-w)S_{\text{bulk}}(T)]$, where w can take any value from the closed interval $[0, 1]$. Note that by setting $w = 0.50$, as we did in all of our runs, we obtain joint likelihood model introduced in Methods.

A.5 Details of input data pre-processing for ALL, TNBC and CRC patients

Below we provide details how bulk data mutation read counts and SCS data mutation matrices, required as the input in our analysis, were obtained for the two ALL, one TNBC real and two CRC data samples analysed in this work.

Pre-processing of ALL data

Raw sequencing data for both ALL patients are available from Sequence Read Archive database under accession no. SRP044380.

In the original study [2], for mutations detected in bulk sample only the fractions of reads supporting the variant allele were reported. Therefore, in order to obtain variant and total read counts for each mutation, we performed mutation calling from raw bulk sequencing data using the pipeline described in [2].

For both patients, SCS mutation matrices were made available in [2].

Pre-processing of TNBC data

Raw bulk and SCS data for this patient are available from Sequence Read Archive database under accession no. SRA053195.

Similarly as for ALL patients, we obtained bulk data read counts from raw bulk sequencing data by the use of the pipeline described in [3].

In contrast to ALL patients, in this case SCS data mutation matrix was not readily available from the original study and we obtained it from raw SCS data using Monovar [4]. All of the parameters used to run this mutation caller, specifically designed for calling single-nucleotide variants from SCS data, are same as in [5].

During the input data preparation we discarded mutations in genes *PTEN* and *TBX3* due to the presence of copy number aberrations, whereas mutation in gene *ECM2*, which is also among mutations selected in the original study (Figure 3(d) in [3]), was discarded due to mislabelling. Namely, the corresponding mutation can not be found in the Supplementary Table 6 in [3] where genomic coordinates required to properly identify mutations were provided. In order to better emphasize major differences between trees reconstructed by SCITE and B-SCITE, we also opted to discard highly uninformative clonal mutation in gene *ARAF* which has marginal effect on the reconstructed trees of tumour evolution and gets assigned as the very first mutation in each of the optimal trees of tumour evolution reported by SCITE and B-SCITE.

Pre-processing of CRC data

Single-cell data matrices were obtained directly from the Supplementary Figure 7 of the original study [6]. All cells without any detected mutation were filtered from the input, as they are non-informative for B-SCITE. After this filtering we were left with 72 single cells for CRC1 (CO5) and 86 single cells for CRC2 (CO8).

In order to obtain bulk data read counts (which we could not find in the original study), we first downloaded primary and metastatic aneuploid whole exome sequencing samples for each patient (raw data available at SRA, runs: SRR3472569, SRR3472571, SRR3472800 and SRR3472796). This was followed by read alignment (using Bowtie 2 [7]), duplicates removal (using Picard tools) and filtering of reads with mapping quality lower than 40. Since for each mutation of interest, reference and variant nucleotide for its genomic position were provided in [6], number of reads supporting variant and reference alleles could be obtained directly from the read alignment files. Mutations having less than 20 reads in total in each of the primary and metastasis sample were excluded from the analysis.

B Supplementary figures

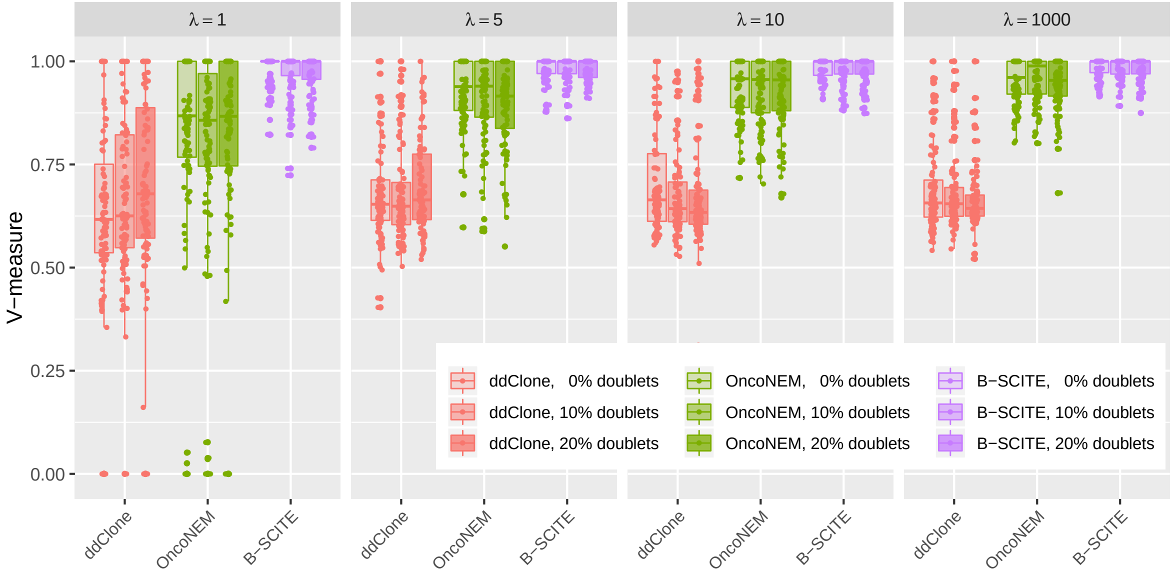

Supplementary Figure 1: Accuracy of mutation clustering by ddClone, OncoNEM and B-SCITE for 100 simulated clonal trees with 6 nodes (clones) and 50 mutations. For the single-cell data, we drew 25 genotypes from each clonal tree for various values of parameter λ which controls bias in sampling single-cells from clones (large values of λ indicate a small bias where probability of drawing single cell from a given clone is usually close to its prevalence in the entire tumour cell population). We also added the following noise to the single-cell genotypes: false positive rate 10^{-5}, false negative rate 0.2, missing (NA) rate 0.05 and doublet rates 0, 0.1 and 0.2. Bulk data coverage was set to 10000 and variant read counts drawn from a binomial distribution. We obtained datasets from trees for each parameter combination. A more detailed description of the simulation data is given in Supplementary Section A.1. For the definition of V-measure see [8]. Source data are provided as a Source Data file.

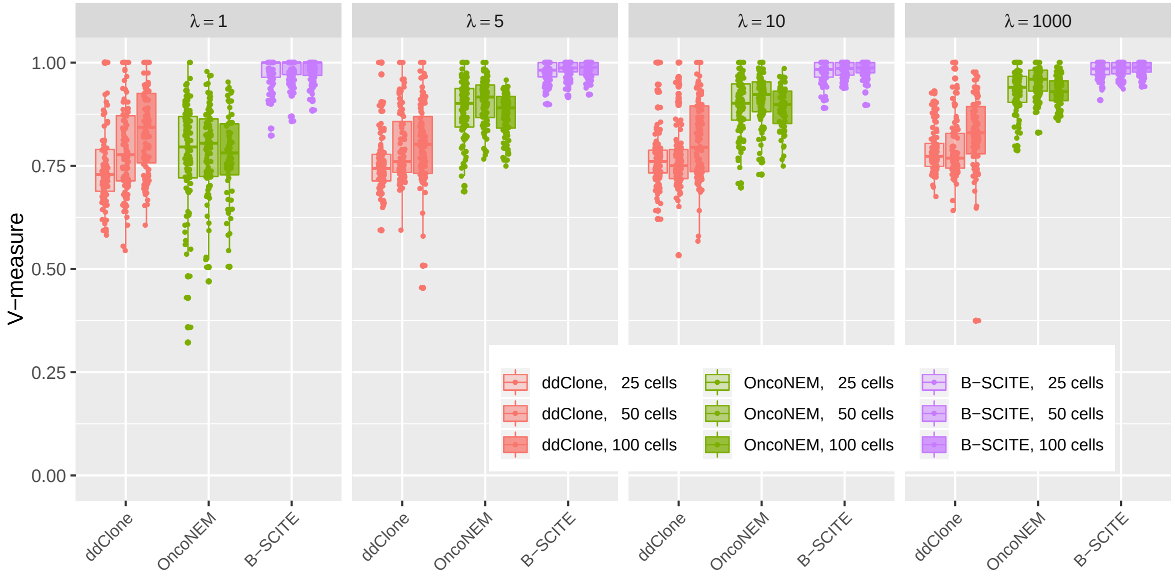

Supplementary Figure 2: Accuracy of mutation clustering by ddClone, OncoNEM and B-SCITE for 100 simulated clonal trees with 10 nodes (clones) and 50 mutations. For the single-cell data, we drew 25, 50 and 100 genotypes from each clonal tree for various values of parameter λ which controls bias in sampling single-cells from clones (large values of λ indicate a small bias where probability of drawing single cell from a given clone is usually close to its prevalence in the entire tumour cell population). We also added the following noise to the single-cell genotypes: false positive rate 10^{-5}, false negative rate 0.2, missing (NA) rate 0.05 and doublet rate 0.1. Bulk data coverage was set to 10000 and variant read counts drawn from a binomial distribution. We obtained datasets from trees for each parameter combination. A more detailed description of the simulation data is given in Supplementary Section A.1. For the definition of V-measure see [8]. Source data are provided as a Source Data file.

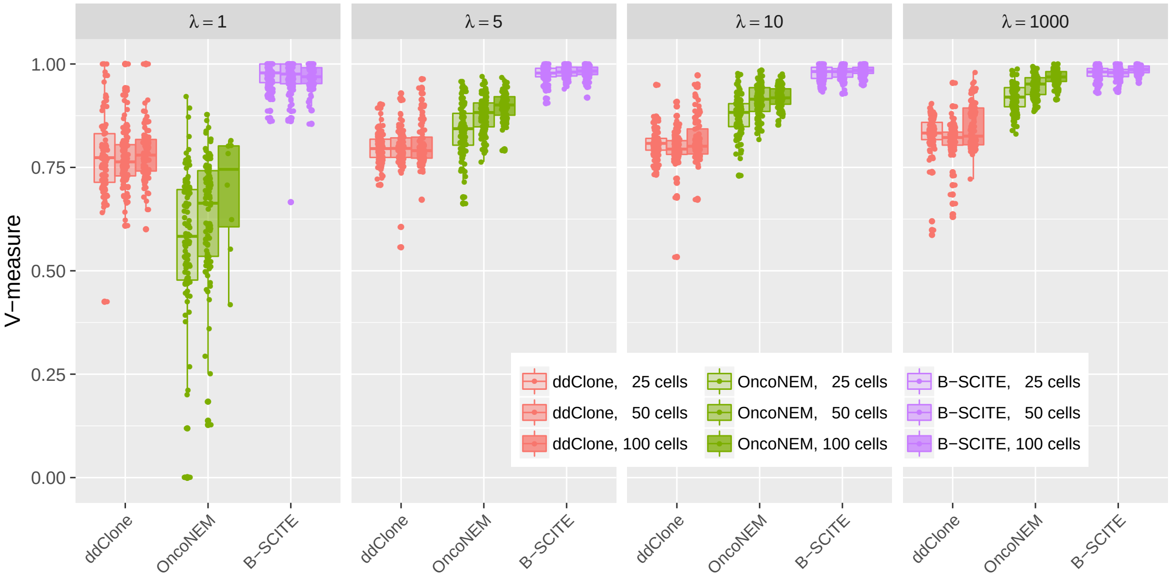

Supplementary Figure 3: Accuracy of mutation clustering by ddClone, OncoNEM and B-SCITE for 100 simulated clonal trees with 20 nodes (clones) and 100 mutations. For the single-cell data, we drew 25, 50 and 100 genotypes from each clonal tree for various values of parameter λ which controls bias in sampling single-cells from clones (large values of λ indicate a small bias where probability of drawing single cell from a given clone is usually close to its prevalence in the entire tumour cell population). We also added the following noise to the single-cell genotypes: false positive rate 10^{-5}, false negative rate 0.2, missing (NA) rate 0.05 and doublet rate 0.1. Bulk data coverage was set to 10000 and variant read counts drawn from a binomial distribution. We obtained datasets from trees for each parameter combination. A more detailed description of the simulation data is given in Supplementary Section A.1. For the definition of V-measure see [8]. Source data are provided as a Source Data file.

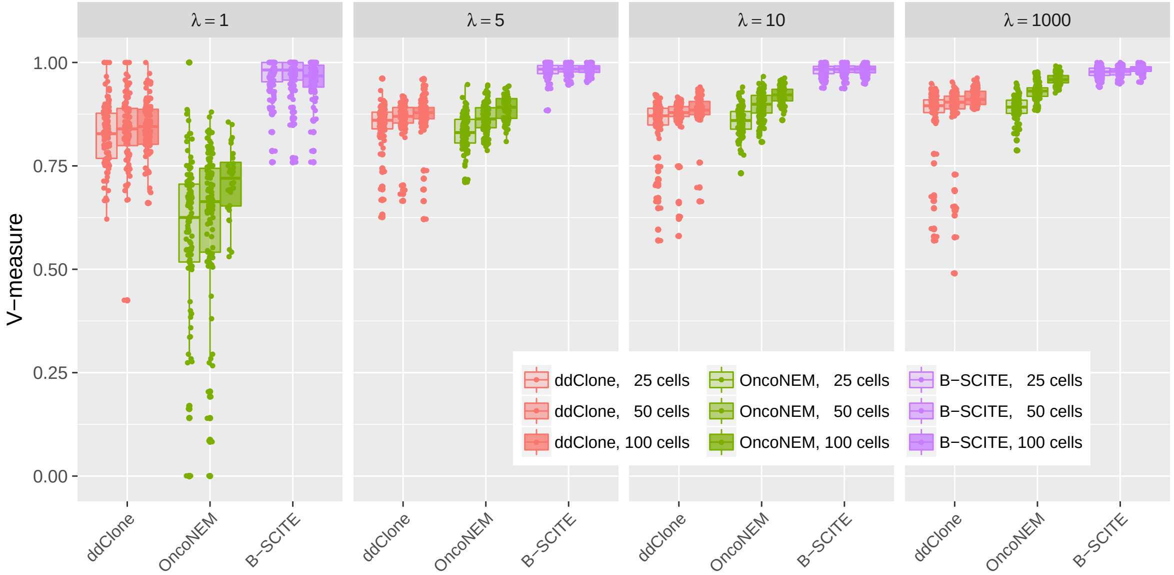

Supplementary Figure 4: Accuracy of mutation clustering by ddClone, OncoNEM and B-SCITE for 100 simulated clonal trees with 40 nodes (clones) and 100 mutations. For the single-cell data, we drew 25, 50 and 100 genotypes from each clonal tree for various values of parameter λ which controls bias in sampling single-cells from clones (large values of λ indicate a small bias where probability of drawing single cell from a given clone is usually close to its prevalence in the entire tumour cell population). We also added the following noise to the single-cell genotypes: false positive rate 10^{-5}, false negative rate 0.2, missing (NA) rate 0.05 and doublet rate 0.1. Bulk data coverage was set to 10000 and variant read counts drawn from a binomial distribution. We obtained datasets from trees for each parameter combination. A more detailed description of the simulation data is given in Supplementary Section A.1. For the definition of V-measure see [8]. Source data are provided as a Source Data file.

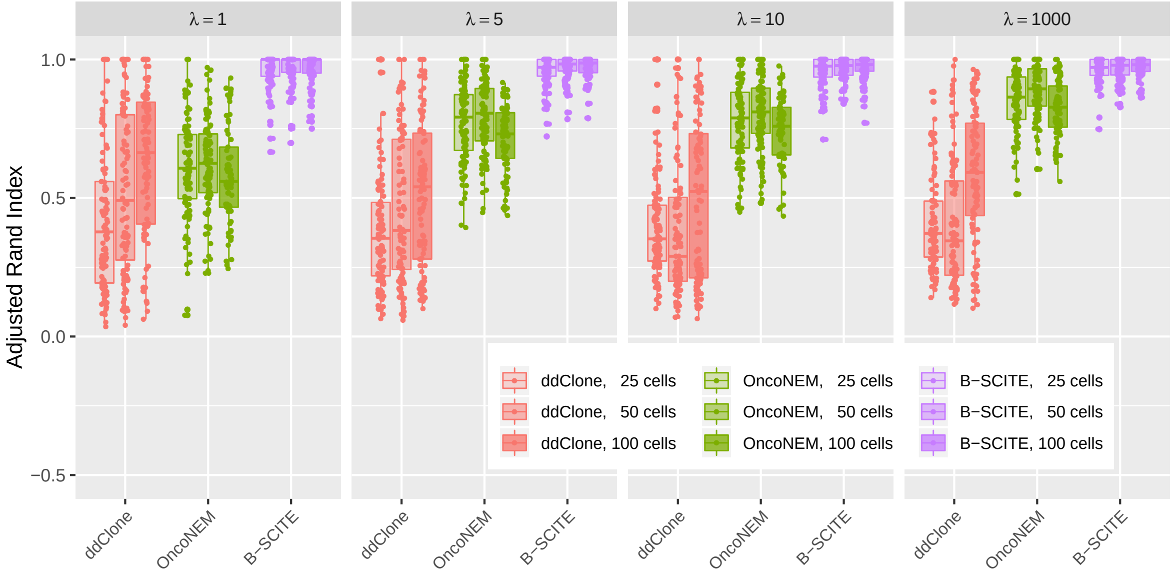

Supplementary Figure 5: Accuracy of mutation clustering in terms of the adjusted Rand index of ddClone, OncoNEM and B-SCITE for 100 simulated clonal trees with 10 nodes (clones) and 50 mutations. The simulation data is the same as for Supplementary Figure 2. Source data are provided as a Source Data file.

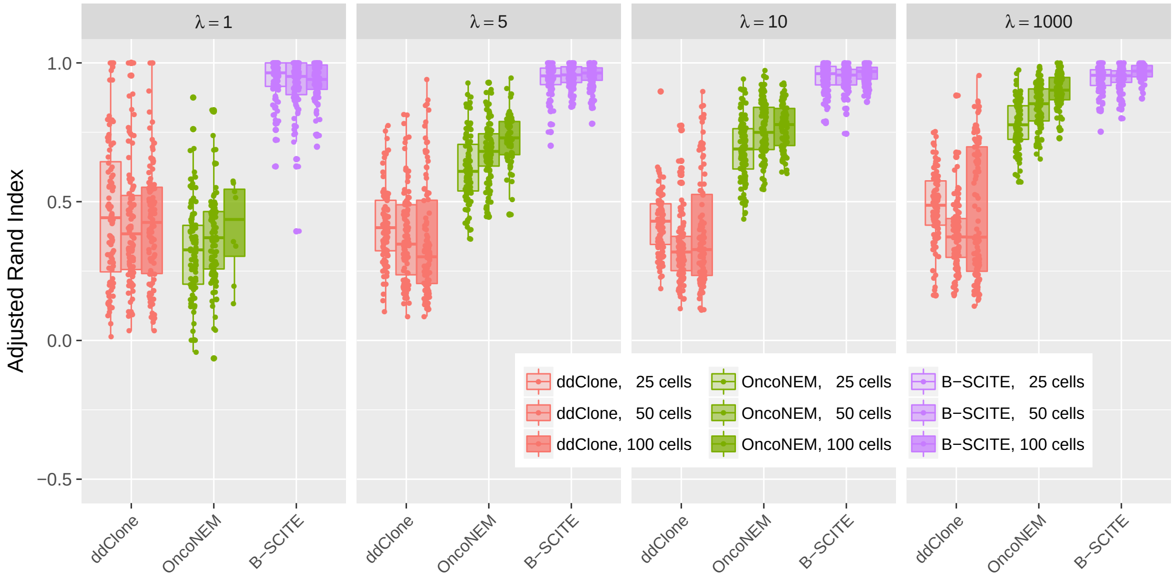

Supplementary Figure 6: Accuracy of mutation clustering in terms of the adjusted Rand index of ddClone, OncoNEM and B-SCITE for 100 simulated clonal trees with 20 nodes (clones) and 100 mutations. The simulation data is the same as for Supplementary Figure 3. Source data are provided as a Source Data file.

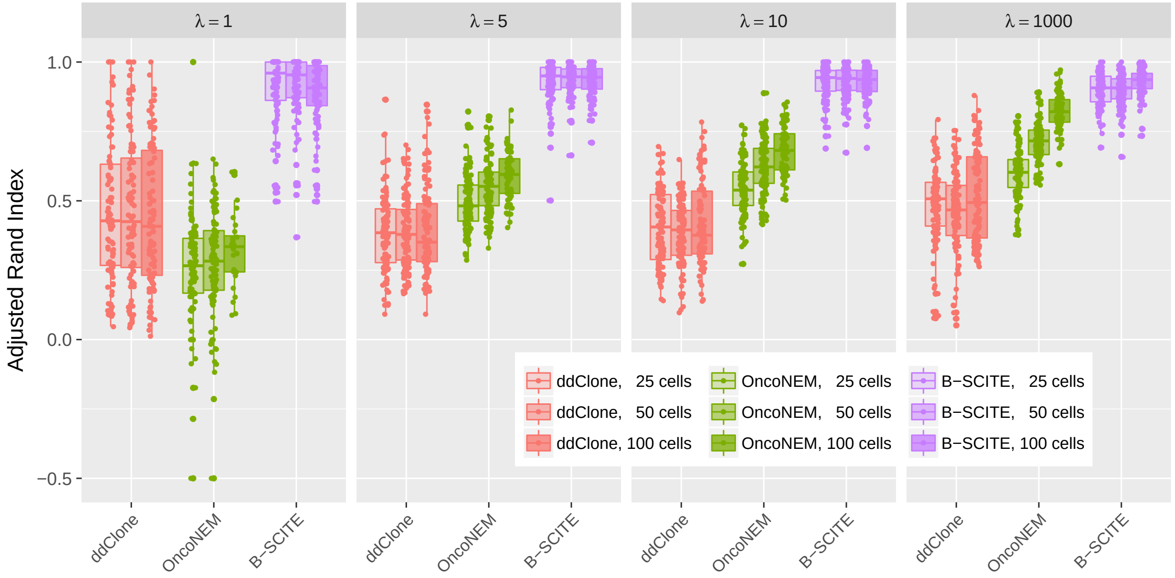

Supplementary Figure 7: Accuracy of mutation clustering in terms of the adjusted Rand index of ddClone, OncoNEM and B-SCITE for 100 simulated clonal trees with 40 nodes (clones) and 100 mutations. The simulation data is the same as for Supplementary Figure 4. Source data are provided as a Source Data file.

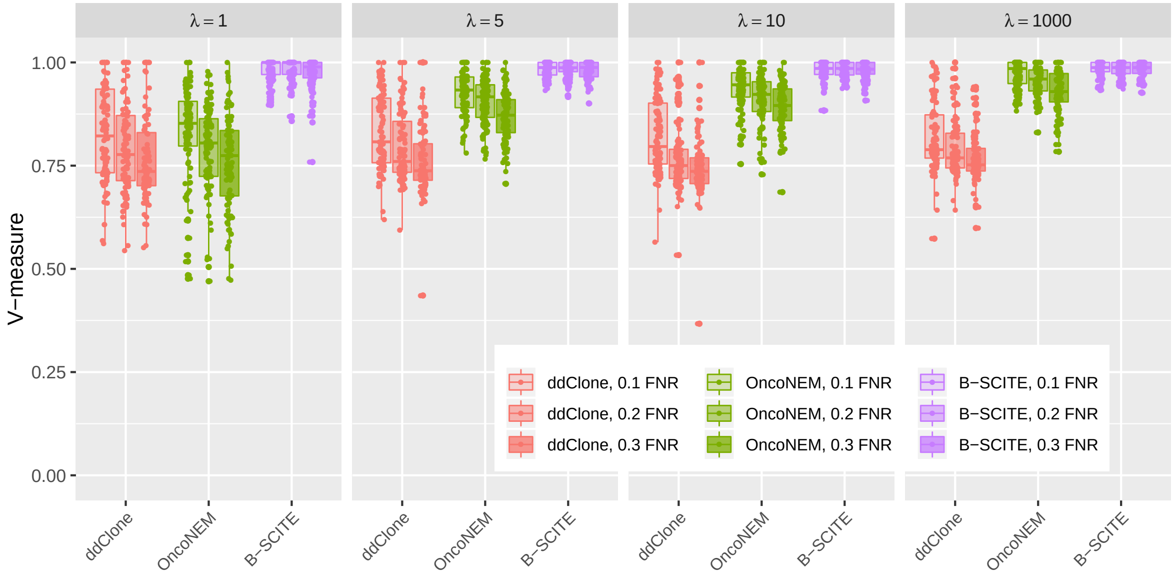

Supplementary Figure 8: Accuracy of mutation clustering by ddClone, OncoNEM and B-SCITE as a function of the false negative rate (FNR). For 100 simulated clonal trees with 10 nodes (clones), 50 mutations, bulk coverage of 10000 and 50 cells, we added false negatives at either 10, 20 or 30%. We also added false positives at a rate of 10^{-5}, missing (NA) data at a rate of 0.05 and doublets with a rate 0.1. Source data are provided as a Source Data file.

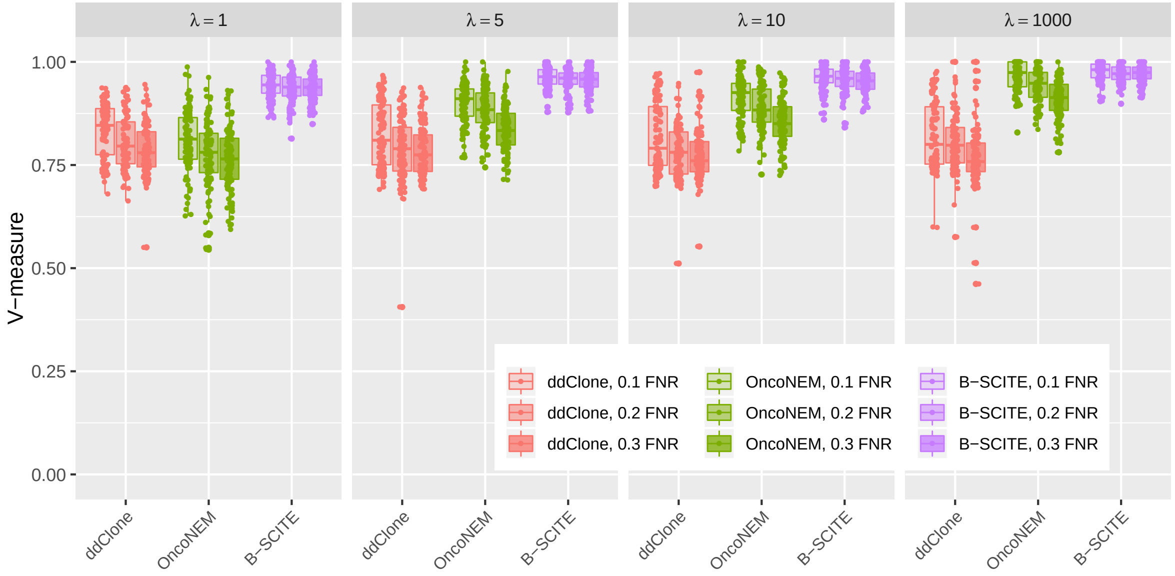

Supplementary Figure 9: Accuracy of mutation clustering by ddClone, OncoNEM and B-SCITE as a function of the false negative rate (FNR). The simulation setting is identical to Supplementary Figure 8, but with a highly elevated false postive rate of 1%. Source data are provided as a Source Data file.

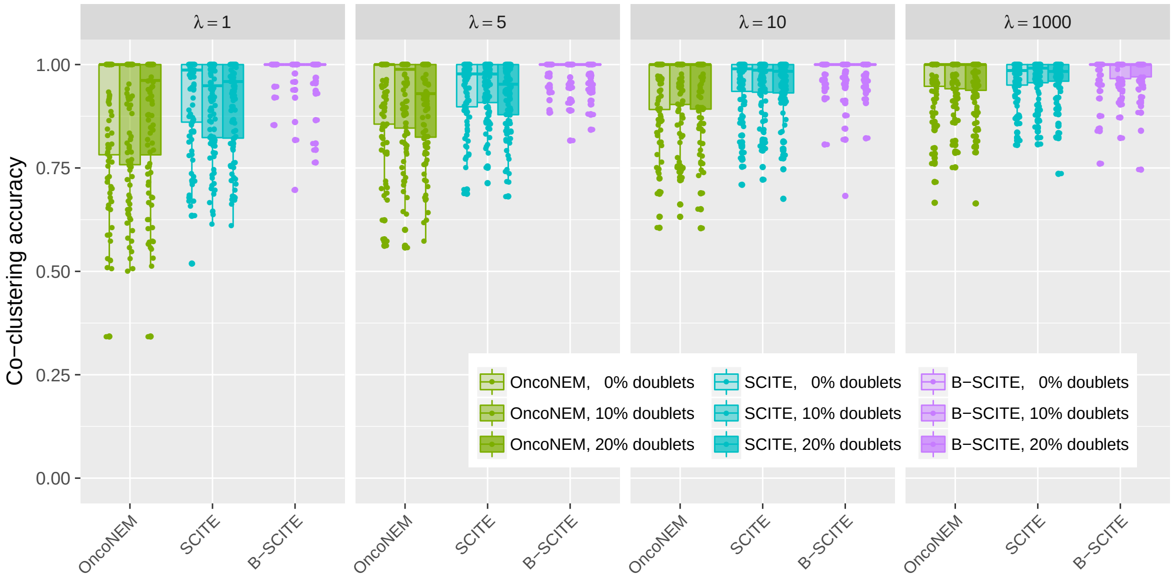

Supplementary Figure 10: Comparison of phylogenetic inference for OncoNEM, SCITE and B-SCITE for 100 simulated clonal trees with 6 nodes (clones) and 50 mutations. For the single-cell data, we drew 25 genotypes from each clonal tree for various values of parameter λ which controls bias in sampling single-cells from clones (large values of λ indicate a small bias where probability of drawing single cell from a given clone is usually close to its prevalence in the entire tumour cell population). We also added the following noise to the single-cell genotypes: false positive rate 10^{-5}, false negative rate 0.2, missing (NA) rate 0.05 and doublet rates 0, 0.1 and 0.2. Bulk data coverage was set to 10000 and variant read counts drawn from a binomial distribution. We obtained datasets from trees for each parameter combination. A more detailed description of the simulation data is given in Supplementary Section A.1. For the definition of Co-clustering accuracy measure see Supplementary Section A.2. Source data are provided as a Source Data file.

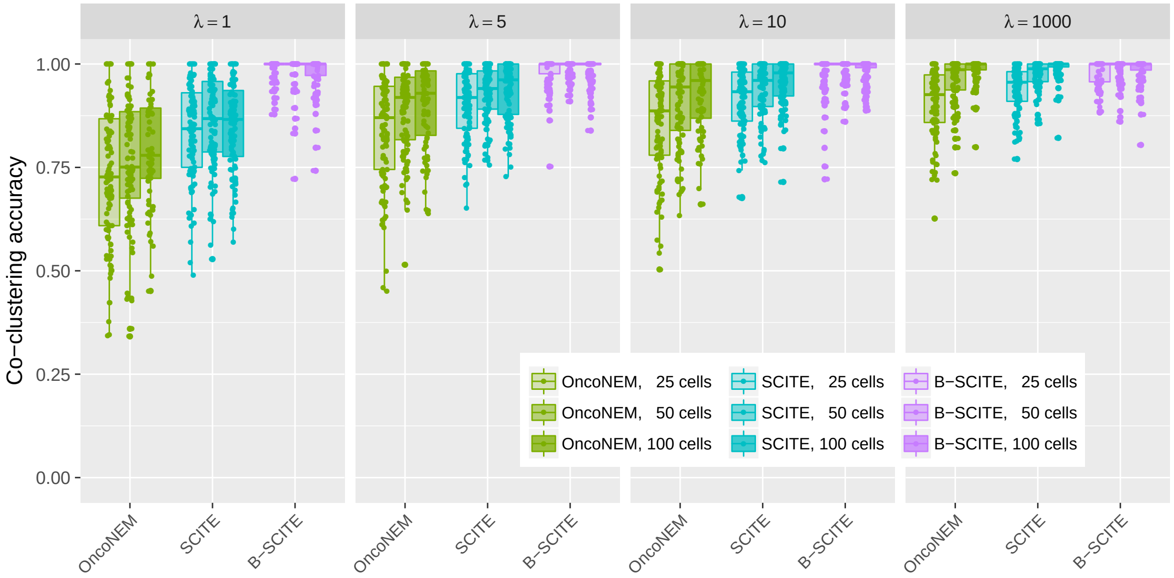

Supplementary Figure 11: Comparison of phylogenetic inference for OncoNEM, SCITE and B-SCITE for 100 simulated clonal trees with 10 nodes (clones) and 50 mutations. For the single-cell data, we drew 25, 50 and 100 genotypes from each clonal tree for various values of parameter λ which controls bias in sampling single-cells from clones (large values of λ indicate a small bias where probability of drawing single cell from a given clone is usually close to its prevalence in the entire tumour cell population). We also added the following noise to the single-cell genotypes: false positive rate 10^{-5}, false negative rate 0.2, missing (NA) rate 0.05 and doublet rate 0.1. Bulk data coverage was set to 10000 and variant read counts drawn from a binomial distribution. We obtained datasets from trees for each parameter combination. A more detailed description of the simulation data is given in Supplementary Section A.1. For the definition of Co-clustering accuracy measure see Supplementary Section A.2. Source data are provided as a Source Data file.

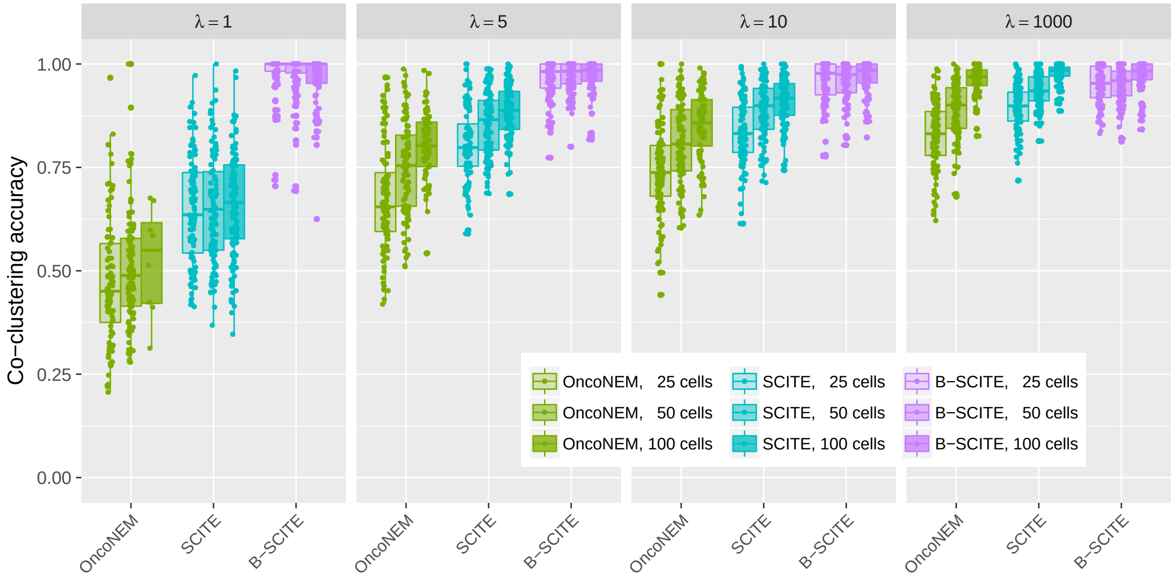

Supplementary Figure 12: Comparison of phylogenetic inference for OncoNEM, SCITE and B-SCITE for 100 simulated clonal trees with 20 nodes (clones) and 100 mutations. For the single-cell data, we drew 25, 50 and 100 genotypes from each clonal tree for various values of parameter λ which controls bias in sampling single-cells from clones (large values of λ indicate a small bias where probability of drawing single cell from a given clone is usually close to its prevalence in the entire tumour cell population). We also added the following noise to the single-cell genotypes: false positive rate 10^{-5}, false negative rate 0.2, missing (NA) rate 0.05 and doublet rate 0.1. Bulk data coverage was set to 10000 and variant read counts drawn from a binomial distribution. We obtained datasets from trees for each parameter combination. A more detailed description of the simulation data is given in Supplementary Section A.1. For the definition of Co-clustering accuracy measure see Supplementary Section A.2. Source data are provided as a Source Data file.

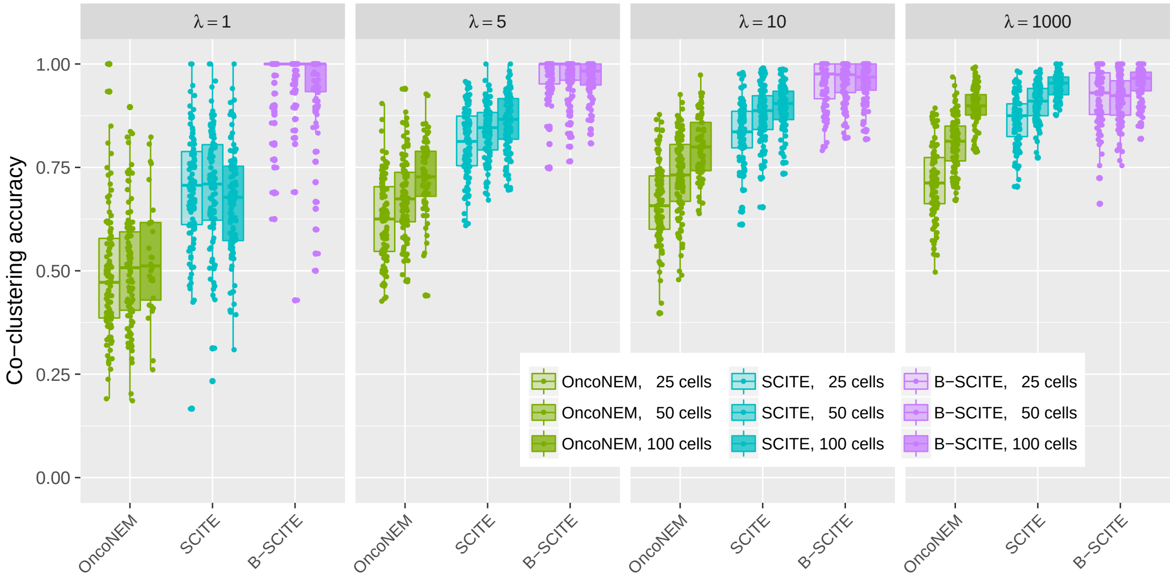

Supplementary Figure 13: Comparison of phylogenetic inference for OncoNEM, SCITE and B-SCITE for 100 simulated clonal trees with 40 nodes (clones) and 100 mutations. For the single-cell data, we drew 25, 50 and 100 genotypes from each clonal tree for various values of parameter λ which controls bias in sampling single-cells from clones (large values of λ indicate a small bias where probability of drawing single cell from a given clone is usually close to its prevalence in the entire tumour cell population). We also added the following noise to the single-cell genotypes: false positive rate 10^{-5}, false negative rate 0.2, missing (NA) rate 0.05 and doublet rate 0.1. Bulk data coverage was set to 10000 and variant read counts drawn from a binomial distribution. We obtained datasets from trees for each parameter combination. A more detailed description of the simulation data is given in Supplementary Section A.1. For the definition of Co-clustering accuracy measure see Supplementary Section A.2. Source data are provided as a Source Data file.

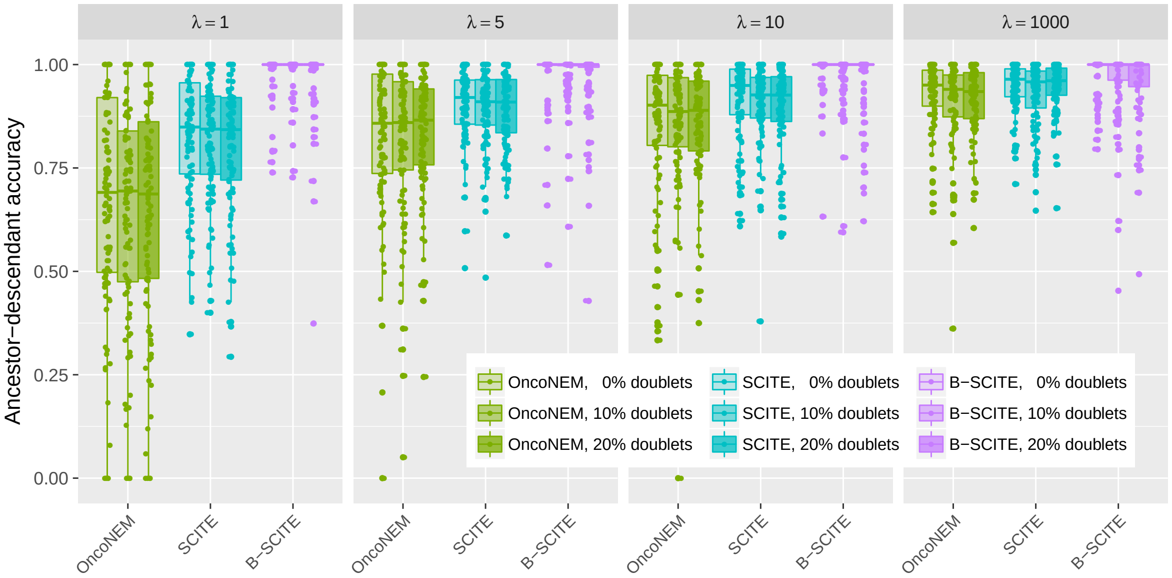

Supplementary Figure 14: Comparison of phylogenetic inference for OncoNEM, SCITE and B-SCITE for 100 simulated clonal trees with 10 nodes (clones) and 50 mutations. For the single-cell data, we drew 25 genotypes from each clonal tree for various values of parameter λ which controls bias in sampling single-cells from clones (large values of λ indicate a small bias where probability of drawing single cell from a given clone is usually close to its prevalence in the entire tumour cell population). We also added the following noise to the single-cell genotypes: false positive rate 10^{-5}, false negative rate 0.2, missing (NA) rate 0.05 and doublet rates 0, 0.1 and 0.2. Bulk data coverage was set to 10000 and variant read counts drawn from a binomial distribution. We obtained datasets from trees for each parameter combination. A more detailed description of the simulation data is given in Supplementary Section A.1. For the definition of Ancestor-descendant accuracy measure see Supplementary Section A.2. Source data are provided as a Source Data file.

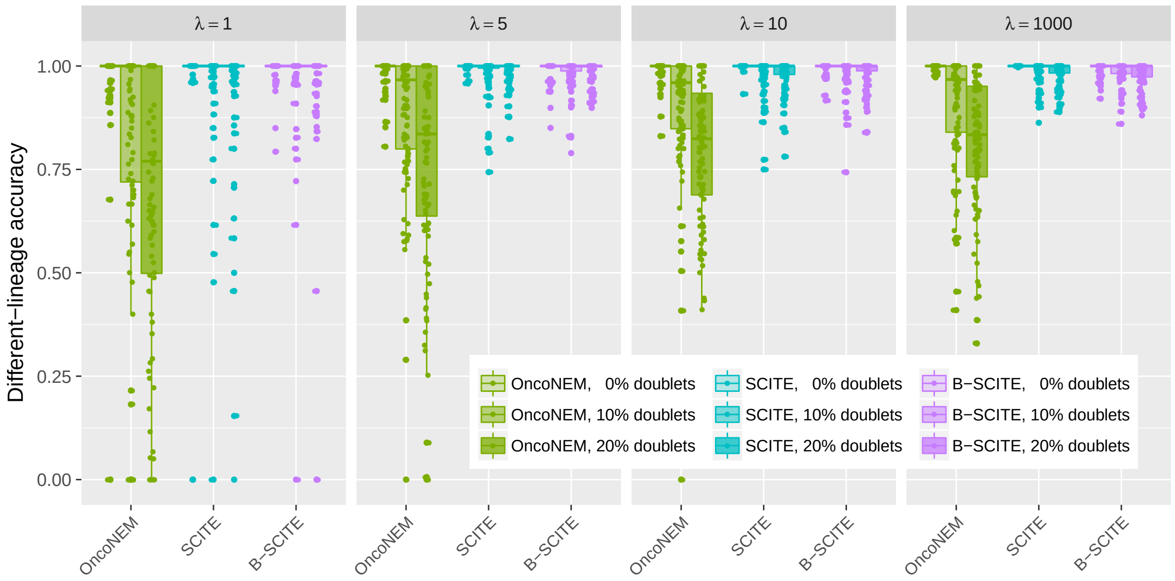

Supplementary Figure 15: Comparison of lineage separation inference for OncoNEM, SCITE and B-SCITE for 100 simulated clonal trees with 10 nodes (clones) and 50 mutations. For the single-cell data, we drew 25 genotypes from each clonal tree for various values of parameter λ which controls bias in sampling single-cells from clones (large values of λ indicate a small bias where probability of drawing single cell from a given clone is usually close to its prevalence in the entire tumour cell population). We also added the following noise to the single-cell genotypes: false positive rate 10^{-5}, false negative rate 0.2, missing (NA) rate 0.05 and doublet rates 0, 0.1 and 0.2. Bulk data coverage was set to 10000 and variant read counts drawn from a binomial distribution. We obtained datasets from trees for each parameter combination. A more detailed description of the simulation data is given in Supplementary Section A.1. For the definition of Different-lineage accuracy measure see Supplementary Section A.2. Source data are provided as a Source Data file.

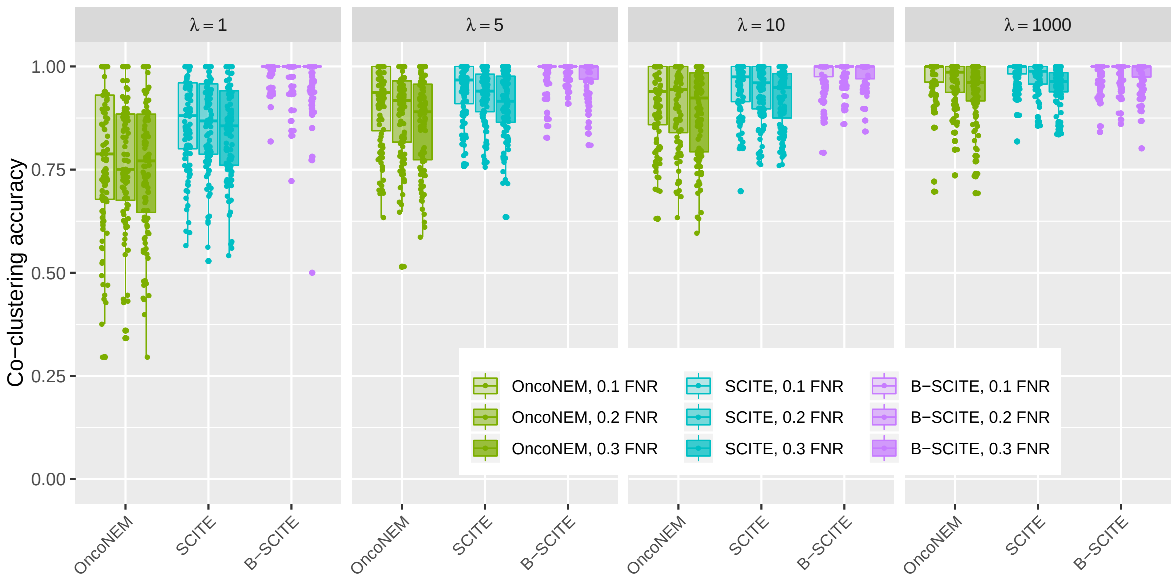

Supplementary Figure 16: Co-clustering accuracy of OncoNEM, SCITE and B-SCITE as a function of the false negative rate (FNR). The simulation is identical to Supplementary Figure 8 with a false positive rate of 10^{-5}. Source data are provided as a Source Data file.

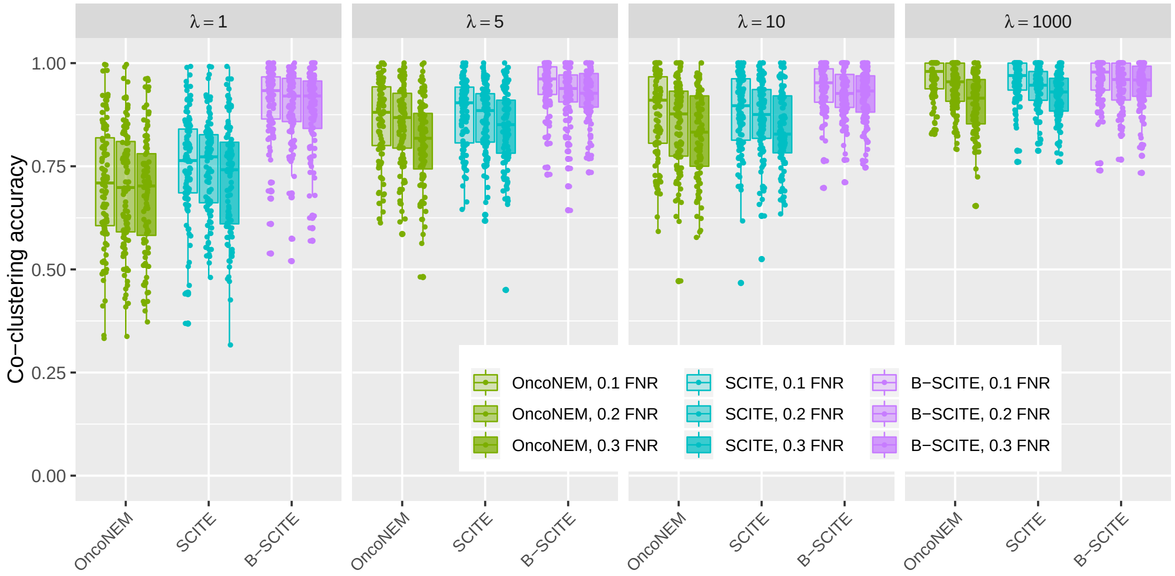

Supplementary Figure 17: Co-clustering accuracy of OncoNEM, SCITE and B-SCITE as a function of the false negative rate (FNR). The simulation is identical to Supplementary Figure 9 with an elevated false positive rate of 1%. Source data are provided as a Source Data file.

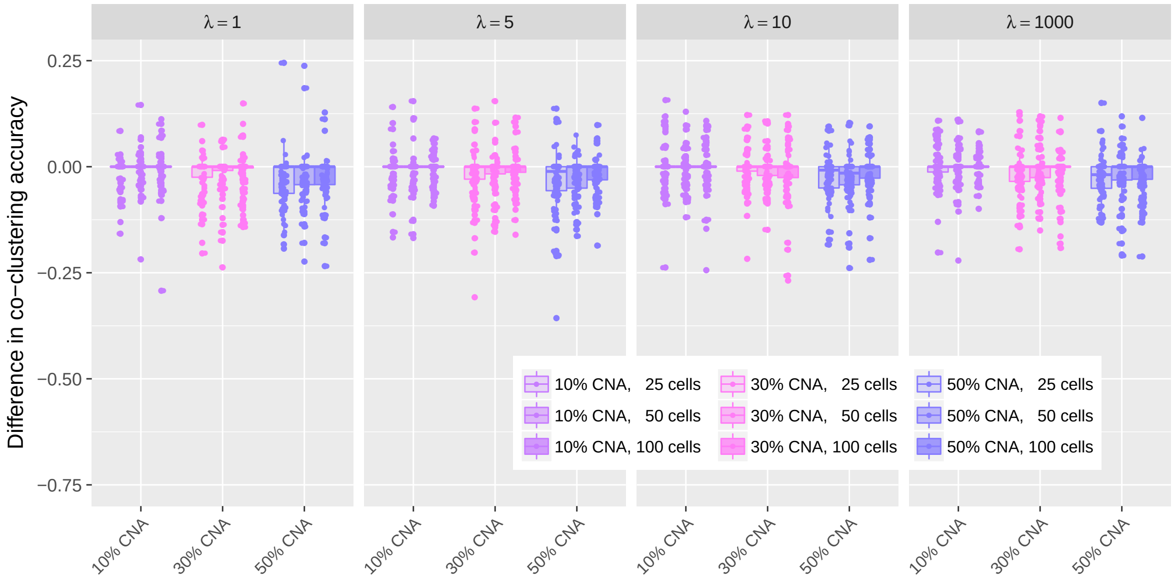

Supplementary Figure 18: The effect of CNAs on the phylogenetic inference of B-SCITE for 100 simulated clonal trees with 10 nodes (clones), 50 mutations and various probabilities (0.1, 0.3 and 0.5) that genomic position of an arbitrary mutation is affected by CNA event. For the single-cell data, we drew 25, 50 and 100 genotypes from each clonal tree for various values of parameter λ which controls bias in sampling single-cells from clones (large values of λ indicate a small bias where probability of drawing single cell from a given clone is usually close to its prevalence in the entire tumour cell population). We also added the following noise to the single-cell genotypes: false positive rate 10^{-5}, false negative rate 0.2, missing (NA) rate 0.05 and doublet rate 0.1. Bulk data coverage was set to 10000 and variant read counts drawn from a binomial distribution. We obtained datasets from trees for each parameter combination. A more detailed description of generating simulated data, including details of simulating CNA events, is given in Supplementary Section A.1. Plotted are the differences in co-clustering accuracy compared to the paired simulation run with no CNAs. For the definition of Co-clustering accuracy measure see Supplementary Section A.2. Source data are provided as a Source Data file.

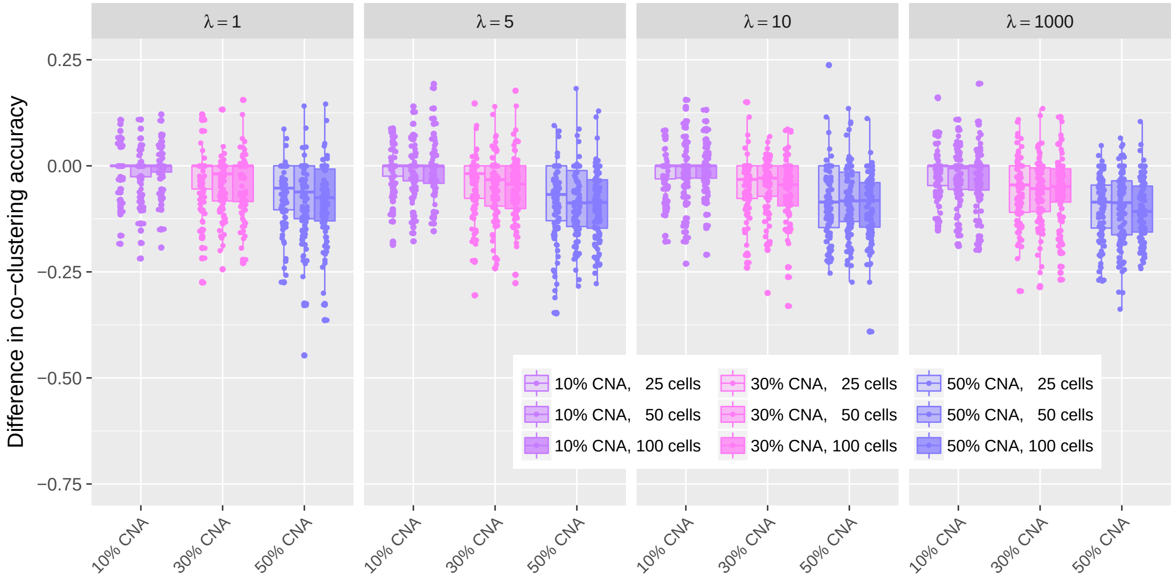

Supplementary Figure 19: The effect of CNAs on the phylogenetic inference of B-SCITE with simulated data as in Supplementary Figure 18 but with a higher coverage of 10^6. Source data are provided as a Source Data file.

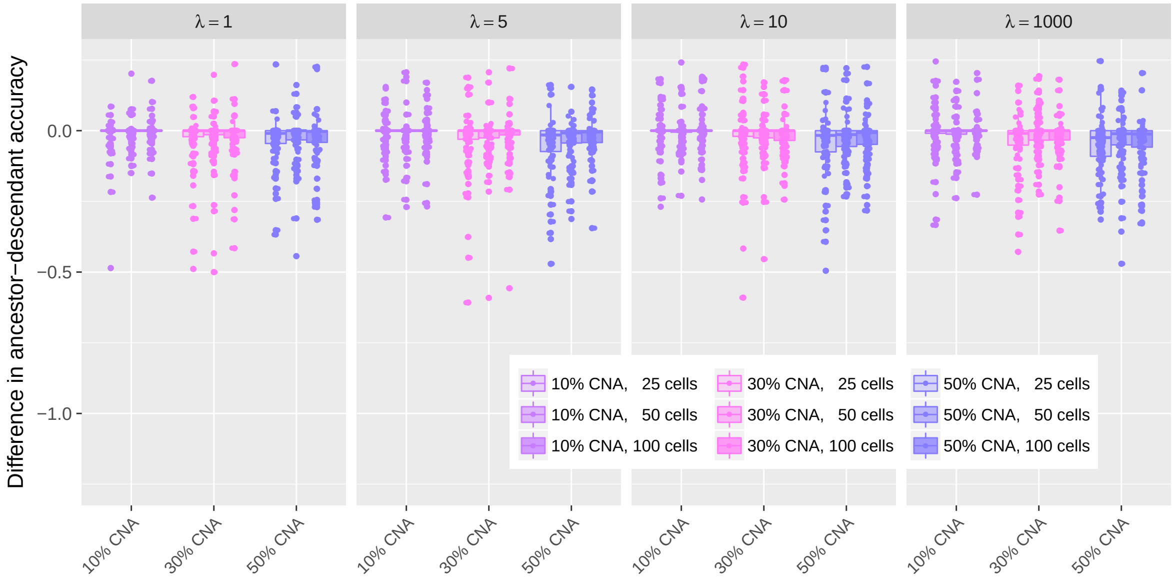

Supplementary Figure 20: The effect of CNAs on the phylogenetic inference of B-SCITE, measured in terms of the ancestor-descendant accuracy for a coverage of 10^4. The simulated data is identical to Supplementary Figure 18. Plotted are the differences in accuracy compared to the paired simulation run with no CNAs. Source data are provided as a Source Data file.

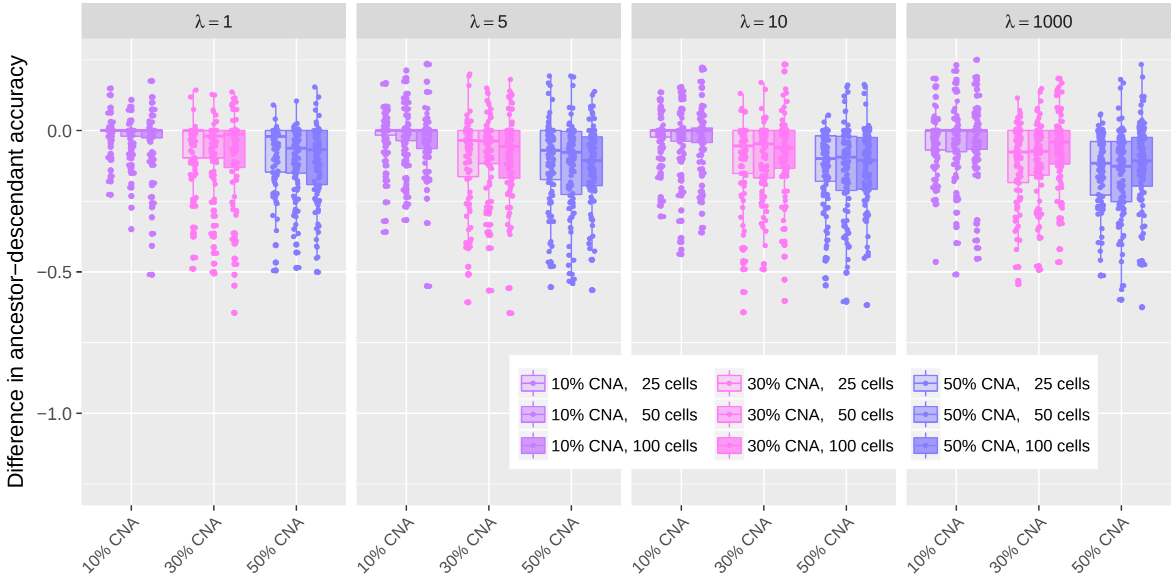

Supplementary Figure 21: The effect of CNAs on the phylogenetic inference of B-SCITE, measured in terms of the ancestor-descendant accuracy for a higher coverage of 10^6. The simulated data is identical to Supplementary Figure 19. Plotted are the differences in accuracy compared to the paired simulation run with no CNAs. Source data are provided as a Source Data file.

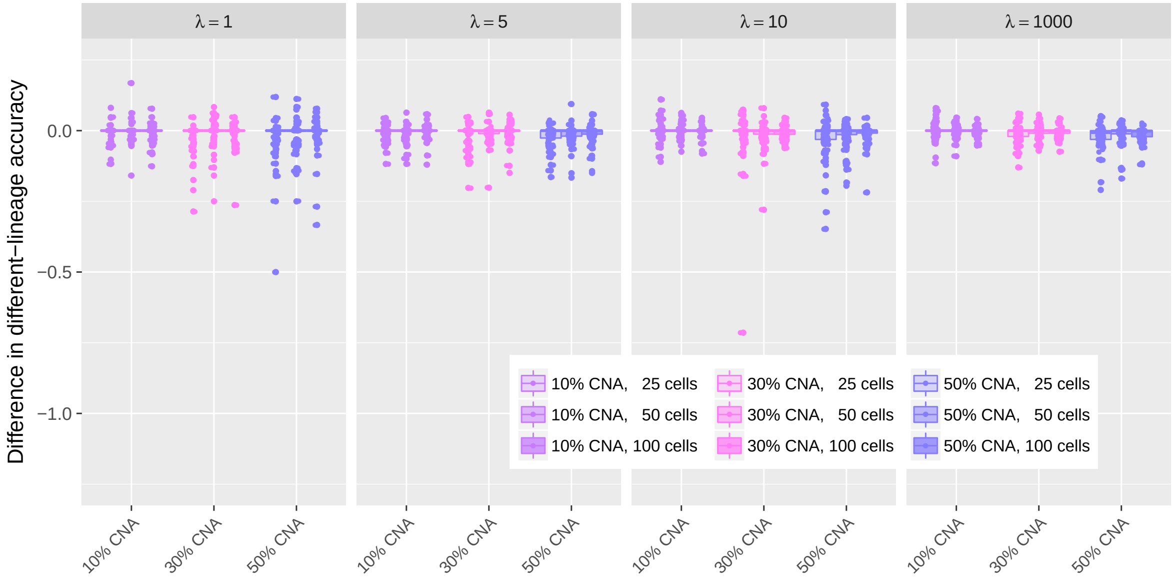

Supplementary Figure 22: The effect of CNAs on the phylogenetic inference of B-SCITE, measured in terms of the different-lineage accuracy for a coverage of 10^4. The simulated data is identical to Supplementary Figure 18. Plotted are the differences in accuracy compared to the paired simulation run with no CNAs. Source data are provided as a Source Data file.

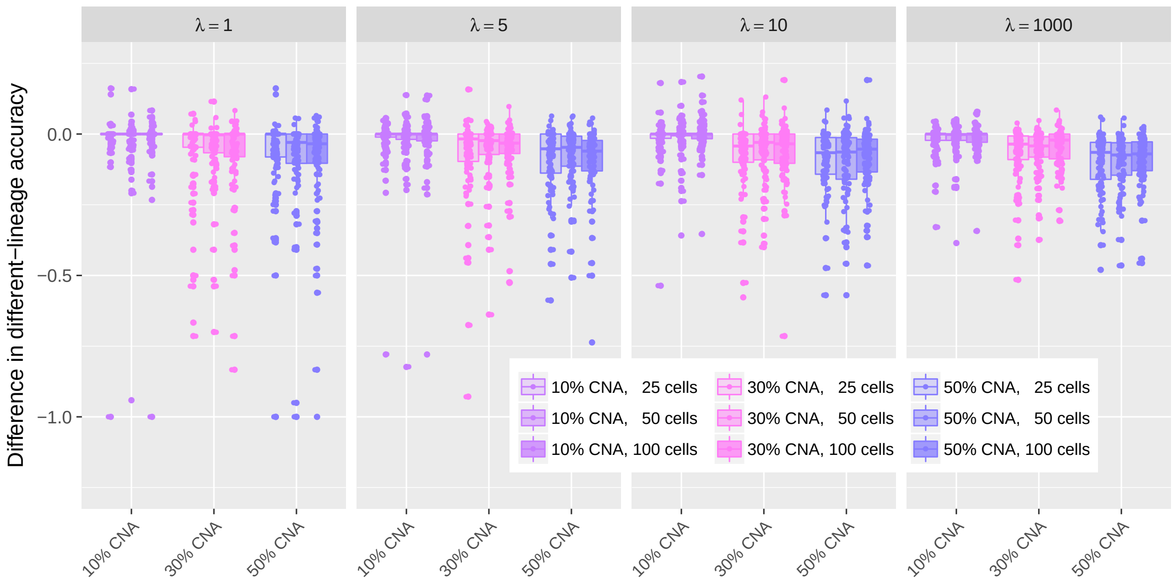

Supplementary Figure 23: The effect of CNAs on the phylogenetic inference of B-SCITE, measured in terms of the different-lineage accuracy for a higher coverage of 10^6. The simulated data is identical to Supplementary Figure 19. Plotted are the differences in accuracy compared to the paired simulation run with no CNAs. Source data are provided as a Source Data file.

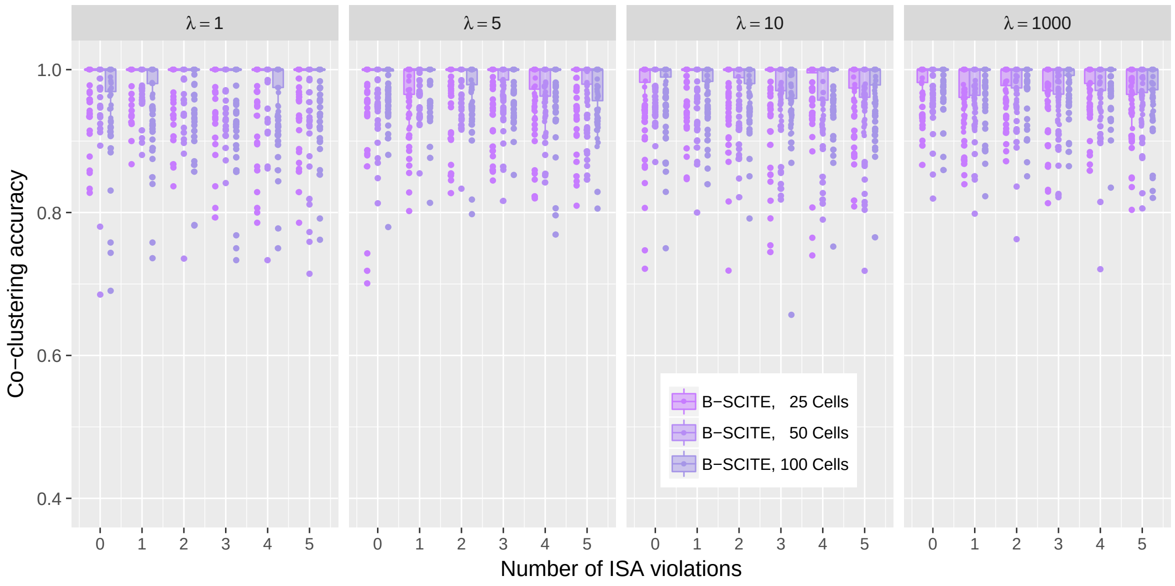

Supplementary Figure 24: The effect of violations of the ISA on the phylogenetic inference of B-SCITE with simulated data with parameters as in Supplementary Figure 18 and an increasing number of violations. The accuracy is computed on the mutations unaffected by violations of the ISA since those affected cannot be uniquely mapped between the generating and inferred trees. Source data are provided as a Source Data file.

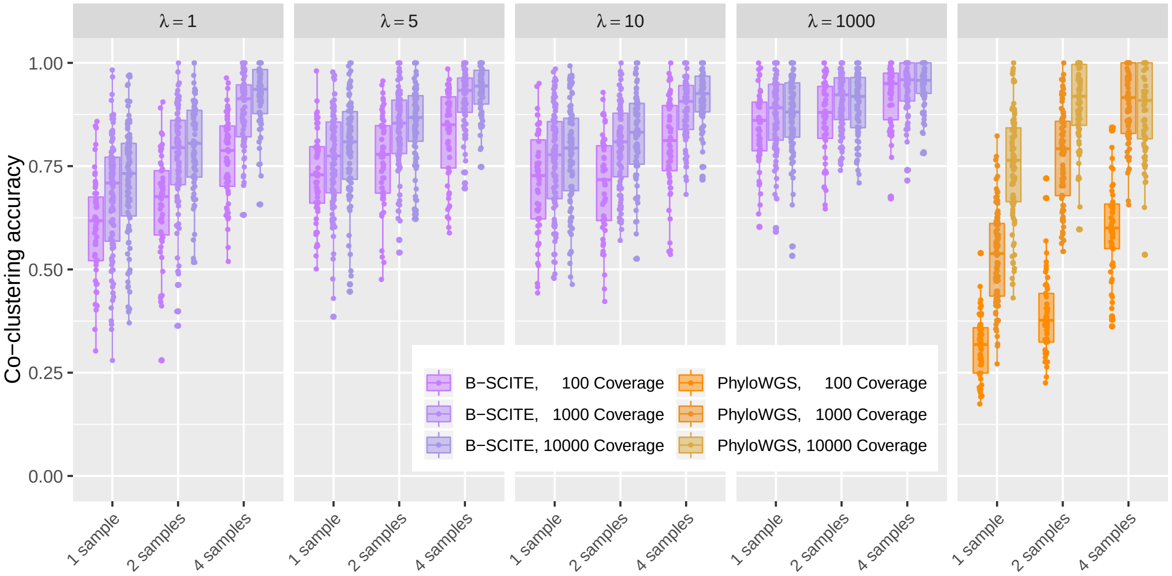

Supplementary Figure 25: The effect of multiple bulk samples and higher bulk coverage on the phylogenetic inference of B-SCITE with simulated data with 10 nodes (clones), 50 mutations a false positive rate of 10^{-5}, a false negative rate of 0.2, a missing (NA) data rate of 0.05 and a doublet rate of 0.1. 25 single cells we samples (with various levels of distortion λ) for various levels of bulk coverage and with 1,2 or 4 bulk samples. The accuracy is computed on all mutations present in the bulk data, including those not sampled in the single cells. Source data are provided as a Source Data file.

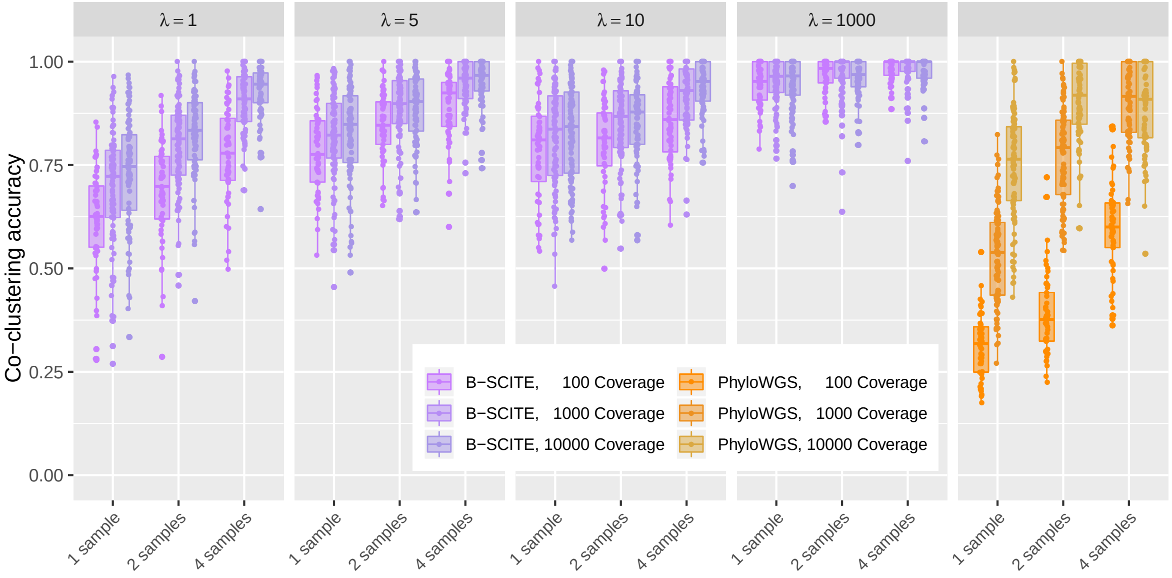

3

Supplementary Figure 26: The effect of multiple bulk samples and higher bulk coverage on the phylogenetic inference of B-SCITE with simulated data as in Supplementary Figure 25 but with 50 cells. The accuracy is computed on all mutations present in the bulk data, including those not sampled in the single cells. Source data are provided as a Source Data file.

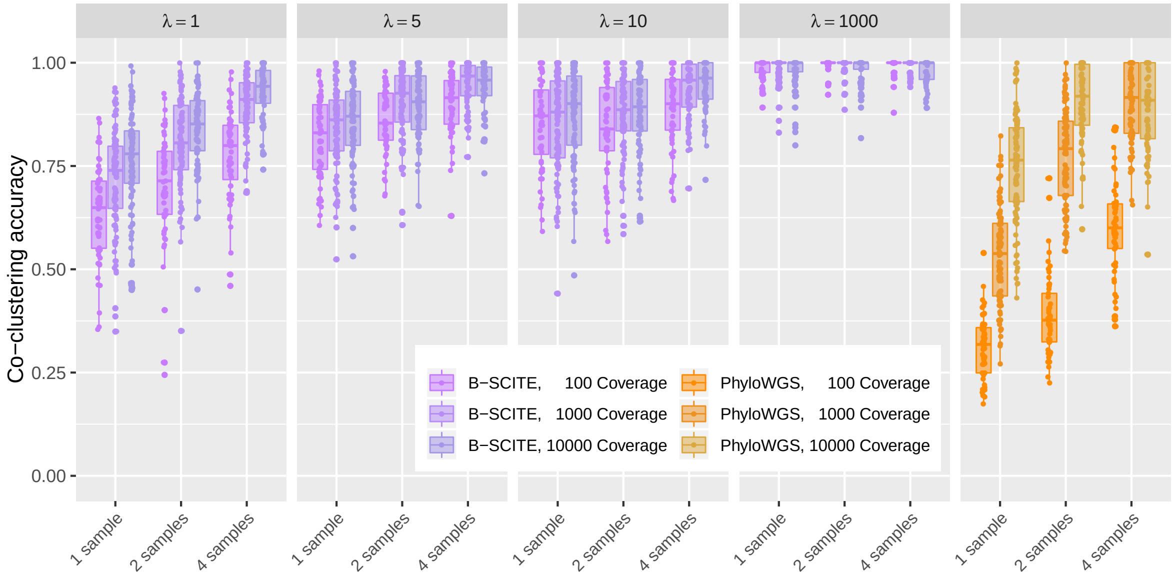

Supplementary Figure 27: The effect of multiple bulk samples and higher bulk coverage on the phylogenetic inference of B-SCITE with simulated data as in Supplementary Figure 25 but with 100 cells. The accuracy is computed on all mutations present in the bulk data, including those not sampled in the single cells. Source data are provided as a Source Data file.

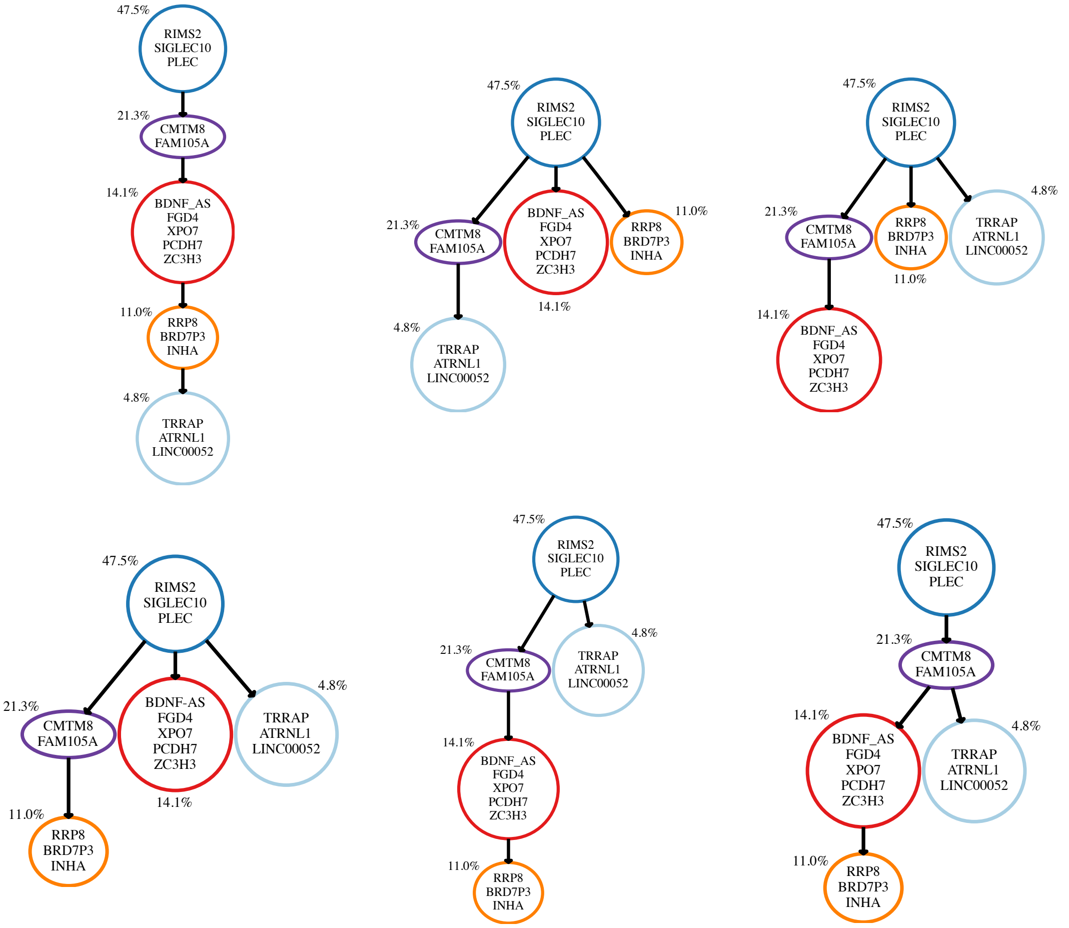

Supplementary Figure 28: Subset of clonal trees inferred with CTPsingle for patient 2 of the leukaemia dataset in [2]: The variant allele frequencies of the five clusters are compatible with multiple different tree topologies and for some topologies multiple optimal assignments of clusters to nodes exist.

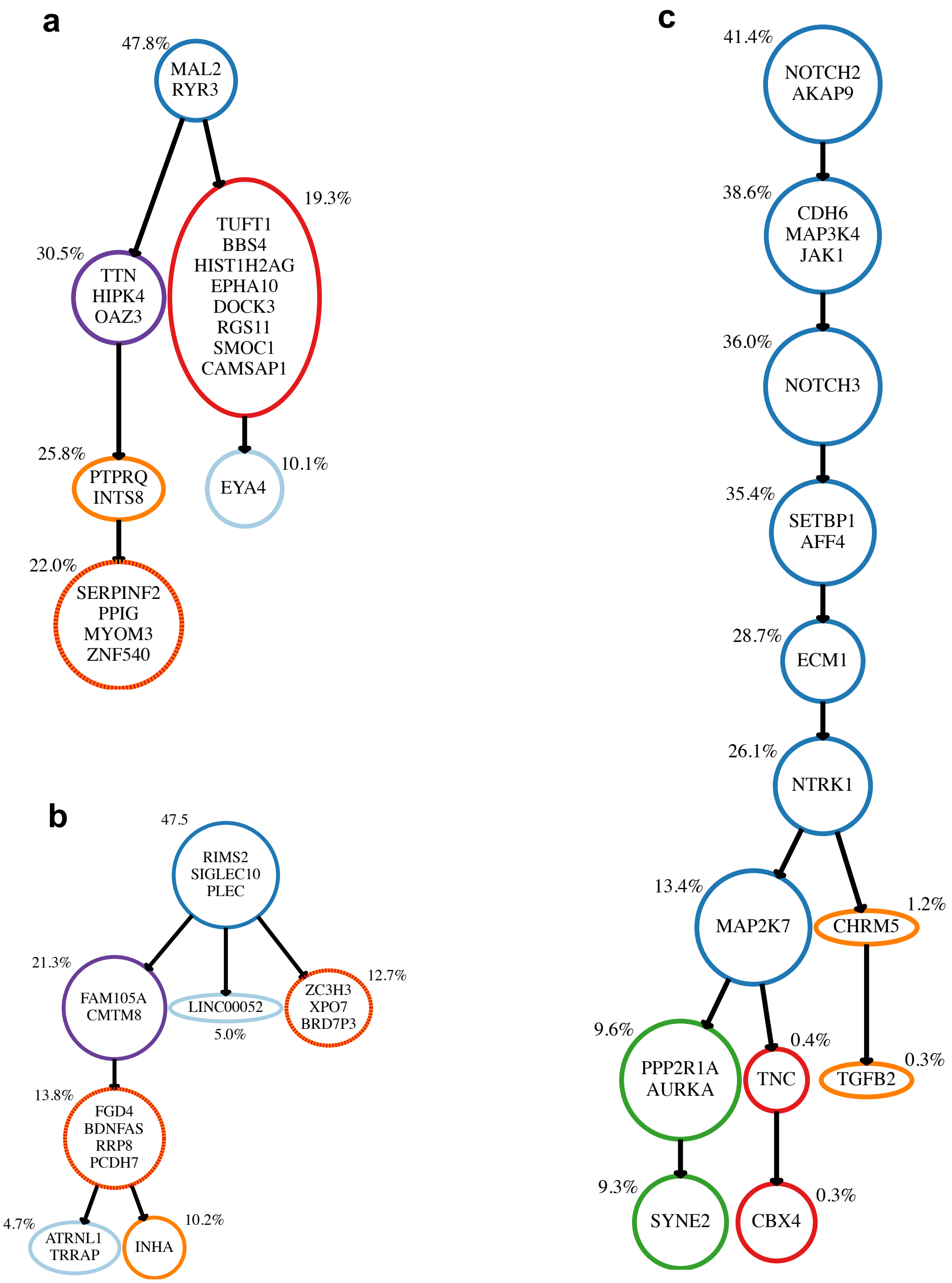

Supplementary Figure 29: Clonal trees derived from B-SCITE mutation trees by node compression: (a) Clonal tree for ALL patient 1 [2](b) Clonal tree for ALL patient 2 [2] (c) Clonal tree for triple negative breast cancer [3].

References

[1] Salehi, S. *et al.* ddClone: joint statistical inference of clonal populations from single cell and bulk tumour sequencing data. *Genome Biology* **18**, 44 (2017).

[2] Gawad, C., Koh, W. & Quake, S. R. Dissecting the clonal origins of childhood acute lymphoblastic leukemia by single-cell genomics. *Proceedings of the National Academy of Sciences* **111**, 17947–17952 (2014).

[3] Wang, Y. *et al.* Clonal evolution in breast cancer revealed by single nucleus genome sequencing. *Nature* **512**, 155 (2014).

[4] Zafar, H., Wang, Y., Nakhleh, L., Navin, N. & Chen, K. Monovar: single-nucleotide variant detection in single cells. *Nature methods* **13**, 505 (2016).

[5] Singer, J., Kuipers, J., Jahn, K. & Beerenwinkel, N. Sciϕ: Single-cell mutation identification via phylogenetic inference. *bioRxiv* 290908 (2018).

[6] Leung, M. L. *et al.* Single cell dna sequencing reveals a late-dissemination model in metastatic colorectal cancer. *Genome research* gr–209973 (2017).

[7] Langmead, B. & Salzberg, S. L. Fast gapped-read alignment with bowtie 2. *Nature methods* **9**, 357 (2012).

[8] Rosenberg, A. & Hirschberg, J. V-measure: A conditional entropy-based external cluster evaluation measure. In *EMNLP-CoNLL*, vol. 7, 410–420 (2007).
