# Supplementary material for: Integrative inference of subclonal tumour evolution from single-cell and bulk sequencing data
Source: Nat Commun. 2019 Jun 21;10:2750. doi: 10.1038/s41467-019-10737-5 (PMC6588593; doi:10.1038/s41467-019-10737-5)
Supplement: Supplementary file 3 — Reporting Summary [file 41467_2019_10737_MOESM3_ESM.pdf]

nature research

Corresponding author(s): Niko Beerenwinkel

Last updated by author(s): May 16, 2019

Reporting Summary

Nature Research wishes to improve the reproducibility of the work that we publish. This form provides structure for consistency and transparency in reporting. For further information on Nature Research policies, see Authors & Referees and the Editorial Policy Checklist.

Statistics

For all statistical analyses, confirm that the following items are present in the figure legend, table legend, main text, or Methods section.

n/a	Confirmed	
☐	☒	The exact sample size (*n*) for each experimental group/condition, given as a discrete number and unit of measurement
☐	☒	A statement on whether measurements were taken from distinct samples or whether the same sample was measured repeatedly
☒	☐	The statistical test(s) used AND whether they are one- or two-sided<br>*Only common tests should be described solely by name; describe more complex techniques in the Methods section.*
☒	☐	A description of all covariates tested
☒	☐	A description of any assumptions or corrections, such as tests of normality and adjustment for multiple comparisons
☒	☐	A full description of the statistical parameters including central tendency (e.g. means) or other basic estimates (e.g. regression coefficient) AND variation (e.g. standard deviation) or associated estimates of uncertainty (e.g. confidence intervals)
☒	☐	For null hypothesis testing, the test statistic (e.g. *F*, *t*, *r*) with confidence intervals, effect sizes, degrees of freedom and *P* value noted<br>*Give P values as exact values whenever suitable.*
☐	☒	For Bayesian analysis, information on the choice of priors and Markov chain Monte Carlo settings
☒	☐	For hierarchical and complex designs, identification of the appropriate level for tests and full reporting of outcomes
☒	☐	Estimates of effect sizes (e.g. Cohen's *d*, Pearson's *r*), indicating how they were calculated

Our web collection on statistics for biologists contains articles on many of the points above.

Software and code

Policy information about availability of computer code

Data collection	fastq-dump (https://ncbi.github.io/sra-tools/fastq-dump.html) was used for downloading raw sequencing data from Sequence Read Archive.
Data analysis	Bowtie 2 (Langmead and Salzberg) was used for read mapping of bulk sequencing data, followed by duplicate removal using Picard tools. Monovar (Zafar et al.) was used for calling SNVs in single-cell data of TNBC patient (matrices for ALL and CRC patients were obtained directly from the original studies). Results of B-SCITE (https://github.com/smalikic/B-SCITE) on the real and simulated data were compared against results obtained by the use of SCITE (Jahn et al.), ddClone (Salehi et al.), OncoNEM (Ross et al.), PhyloWGS (Deshwar et al.) and CTPsingle (Donmez et al.).

For manuscripts utilizing custom algorithms or software that are central to the research but not yet described in published literature, software must be made available to editors/reviewers. We strongly encourage code deposition in a community repository (e.g. GitHub). See the Nature Research guidelines for submitting code & software for further information.

Data

Policy information about availability of data

All manuscripts must include a data availability statement. This statement should provide the following information, where applicable:

- Accession codes, unique identifiers, or web links for publicly available datasets
- A list of figures that have associated raw data
- A description of any restrictions on data availability

The human sequencing datasets utilized in this study are available for download from the Sequence Read Archive with the accession numbers: SRP044380 (for ALL patients), SRA053195 (for TNBC patient) and SRP074289 (for CRC patients).

Field-specific reporting

Please select the one below that is the best fit for your research. If you are not sure, read the appropriate sections before making your selection.

☒ Life sciences ☐ Behavioural & social sciences ☐ Ecological, evolutionary & environmental sciences

For a reference copy of the document with all sections, see nature.com/documents/nr-reporting-summary-flat.pdf

Life sciences study design

All studies must disclose on these points even when the disclosure is negative.

Sample size	100 repetitions were chosen for each simulation to give a good idea of the distribution of accuracy measures, while balancing the computational cost
Data exclusions	No data were excluded.
Replication	The developed software produces the same results when run with the same starting conditions/parameters (including the MCMC seed).
Randomization	Does not apply. This work does not describe an experimental study that requires allocation into groups.
Blinding	Does not apply. This work does not describe an experimental study that requires allocation into groups.

Reporting for specific materials, systems and methods

We require information from authors about some types of materials, experimental systems and methods used in many studies. Here, indicate whether each material, system or method listed is relevant to your study. If you are not sure if a list item applies to your research, read the appropriate section before selecting a response.

Materials & experimental systems

n/a	Involved in the study	
☒	☐	Antibodies
☒	☐	Eukaryotic cell lines
☒	☐	Palaeontology
☒	☐	Animals and other organisms
☒	☐	Human research participants
☒	☐	Clinical data

Methods

n/a	Involved in the study	
☒	☐	ChIP-seq
☒	☐	Flow cytometry
☒	☐	MRI-based neuroimaging
